# Transformer-based deep learning model for real-time prediction of intraoperative hypotension using dynamic time-series vital signs: A retrospective study

**DOI:** 10.1371/journal.pmed.1005024

**Published:** 2026-03-25

**Authors:** Shouqiang Zhu, Weihai Shi, Haichuan Qian, Xiaoqiang Tong, Rongliang Hu, Jinhua Bo, Xiaoping Gu

**Affiliations:** 1 Department of Anesthesiology, Nanjing Drum Tower Hospital, Affiliated Hospital of Medical School, Nanjing University, Nanjing, China; 2 Nanjing Qicheng Medical Technology Co., Ltd., Nanjing, China; Liverpool John Moores University - City Campus: Liverpool John Moores University, UNITED KINGDOM OF GREAT BRITAIN AND NORTHERN IRELAND

## Abstract

**Background:**

The clinical importance of transient intraoperative hypotension (IOH) remains debated, and existing models often rely on high-resolution waveform data that are not routinely available.

**Methods and findings:**

We developed a Transformer-based deep learning model to predict IOH in real time using continuous vital sign time-series data. The model was trained on 319,699 surgical cases from a tertiary hospital in China (2013–2023) and externally validated using an independent dataset from South Korea. Model interpretability was explored through a real-time alert simulation using 10 representative surgical cases from the internal validation cohort, comparing predicted IOH risk trajectories with measured mean arterial pressure (MAP). To assess clinical relevance, a nested cohort study evaluated the association between IOH burden (cumulative MAP ≤65/60/55 mmHg in mmHg·min) and postoperative acute kidney injury (AKI) and acute kidney disease (AKD). The Transformer model achieved strong prediction performance at 5-, 10-, and 15-min horizons (AUCs 0.904, 0.892, 0.882; recall ≥88.3%). Compared with XGBoost, the Transformer had higher recall (internal 5-min recall 0.891 versus 0.737) and substantially better probability calibration (expected calibration error 0.0083 versus 0.0373). XGBoost showed higher overall accuracy and specificity (internal 5-min specificity 0.913 versus 0.723). External validation confirmed comparable discrimination across models and generalizability, with attenuated calibration differences. In alert simulations, predicted IOH risk closely corresponded to MAP fluctuations. IOH burden was significantly associated with postoperative AKI and AKD (MAP ≤65 mmHg: OR per 60 mmHg·min 1.10 (95% CI, [1.02, 1.19]; *p* = 0.012) for AKI; 1.26 (95% CI, [1.19, 1.33]; *p* < 0.001) for AKD). The study is limited by its retrospective design, and prospective, multicenter validation is needed to confirm real-time applicability and generalizability of the model.

**Conclusions:**

IOH burden is associated with increased risk of postoperative AKI and AKD. The Transformer model prioritizes sensitivity and calibration for short-term IOH prediction, whereas XGBoost emphasizes accuracy and specificity, reflecting different operating characteristics. Prospective, real-time evaluation is needed before clinical implementation.

## Author summary

**Why was this study done?:**

- Intraoperative hypotension (IOH), or low blood pressure during surgery, is common and may increase the risk of kidney injury and other complications.
- Existing prediction tools often rely on complex arterial waveform data that are not routinely available in many operating rooms or real-time monitoring systems.
- We aimed to develop and validate a machine learning model that can predict IOH in real time using routinely collected vital signs.

**What did the researchers do and find?:**

- We developed a Transformer-based deep learning model using more than 300,000 surgical cases from a tertiary hospital in China and evaluated it on an independent external dataset from South Korea.
- The model predicted IOH 5–15 min in advance with high sensitivity. We also compared its performance with an XGBoost model to examine differences in prediction characteristics.
- Predicted IOH risk trajectories corresponded closely with observed mean arterial pressure (MAP) changes, and a higher IOH burden was associated with increased risk of postoperative kidney injury.

**What do these findings mean?:**

- Real-time prediction of IOH may help clinicians intervene earlier and potentially reduce postoperative complications.
- The Transformer model achieved higher sensitivity, whereas the XGBoost model showed higher specificity and overall accuracy. The Transformer also demonstrated better calibration in internal validation, while calibration differences were smaller in the external dataset.
- Because this study was retrospective, prospective validation is needed to confirm real-time clinical utility and generalizability across diverse surgical settings.

## Abstract 3

In this retrospective study, Shouqiang Zhu, Weihai Shi and developed a deep learning model for real-time prediction on intraoperative hypotension using dynamic time-series vital signs from a Chinese cohort.

## Introduction

General anesthesia is a cornerstone of modern surgical practice, enabling safe and painless procedures by inducing reversible unconsciousness and immobility [1]. However, it can significantly alter cardiovascular physiology, often leading to vasodilation and reduced cardiac output. As a result, intraoperative hypotension (IOH)—commonly defined as a mean arterial pressure (MAP) below 65 mmHg sustained for at least 1 min—frequently occurs and is associated with serious postoperative complications, including myocardial injury [2,3], acute renal injury [4,5], and increased mortality [6,7]. Beyond physiological harm, IOH has also been linked to longer hospital stays, higher healthcare costs, and increased resource utilization [8–10], underscoring the critical need for early and accurate prediction to facilitate timely intervention and improve patient outcomes. Despite these associations, the clinical significance of brief or transient IOH episodes remains debated. Several reports question whether short-duration hypotension independently contributes to adverse outcomes after adjusting for comorbidities and surgical factors [11,12]. This ongoing controversy highlights the need for better stratification of IOH risk and more refined predictive tools to identify clinically meaningful hypotensive events.

Existing IOH prediction models often rely on complex physiological data, including invasive arterial waveform monitoring, advanced hemodynamic indices, and multidimensional variables [13–15]. While these models, such as those based on machine learning and feature engineering, have demonstrated promising predictive power, their clinical utility remains constrained by real-time deployment challenges. Among these, the Hypotension Prediction Index (HPI) is one of the most widely adopted models in clinical practice (commercialized as the Acumen HPI software by Edwards Lifesciences, integrated into the HemoSphere monitoring platform). Developed using machine learning applied to high-fidelity arterial waveform features, HPI predicts hypotensive events minutes before they occur [16,17]. Despite its success, its reliance on continuous waveform data limits its applicability in settings where such high-resolution signals are often not available.

To enhance real-world applicability, we developed a deep learning model (Transformer) using only routinely collected time-series vital signs—such as MAP, heart rate, and oxygen saturation—without incorporating detailed intraoperative variables like anesthetic dosing, fluid balance, or surgical complexity. While such factors undoubtedly influence hypotension, their real-time availability is often limited, especially in low-resource or non-research settings. Previous studies have shown that models relying solely on vital signs can achieve comparable or superior predictive performance (AUC >0.90) [18] compared to models with rich clinical inputs (e.g., Lee and colleagues, AUC 0.74) [19], and the dynamic patterns of vital signs may implicitly reflect physiological effects of interventions. Transformer-based architectures, with their capacity to model long-range temporal dependencies, further enhance the ability to capture such interactions without explicitly including these variables. This streamlined design supports scalability and timely intraoperative decision support across diverse surgical contexts. The Transformer model, originally developed for natural language processing tasks, has demonstrated remarkable performance in learning temporal dependencies from sequential data [20–22]. Unlike traditional recurrent neural networks, the Transformer architecture utilizes self-attention mechanisms to model long-range dependencies more effectively and efficiently, making it well-suited for continuous intraoperative monitoring applications [23].

Early prediction of IOH allows anesthesiologists to initiate timely preventive measures, such as fluid optimization, vasopressor administration, or adjustment of anesthetic depth, before a critical blood pressure drop occurs. The 5- to 15-min prediction horizons were selected based on prior studies [14,24] and clinical practice as an actionable window for hemodynamic intervention. Potential trade-offs of these interventions, such as fluid overload or increased cardiac workload, highlight that predictive alerts are intended to support, rather than replace, clinical judgment.

In this study, we developed and validated a Transformer-based deep learning model designed to predict IOH in real time using only intraoperative vital sign time-series data. This approach aimed to achieve high temporal resolution and generalizability while avoiding reliance on high-frequency waveform or medication data that may not be routinely available in clinical settings. To strengthen the clinical relevance of transient IOH, we conducted a nested retrospective cohort analysis to examine the association between hypotension exposure—quantified as the area under threshold for MAP <65, <60, and <55 mmHg—and postoperative AKI and AKD. Furthermore, to demonstrate the clinical interpretability and temporal behavior of model predictions, we randomly selected 10 representative surgical cases from the internal validation cohort for detailed trajectory analysis, presenting both MAP trajectories and predicted IOH risk over time.

## Methods

### Study population

Owing to the retrospective design, the requirement for informed consent was waived. The study was conducted in strict accordance with the original version of the study protocol submitted for approval to the institutional ethics committee. No amendments or deviations from the approved protocol occurred during the study period. We screened all adult patients (aged ≥18 years) who received general anesthesia at Nanjing Drum Tower Hospital—a large tertiary academic medical center—between January 1, 2013, and December 30, 2023. Anesthesia care was provided by both resident and attending anesthesiologists from the Department of Anesthesiology. The VitalDB dataset was obtained from the PhysioNet repository and includes biosignal data collected at Seoul National University Hospital (IRB No. H-1408-101-605; ClinicalTrials.gov Identifier NCT02914444). The secondary use of de-identified data was approved by the IRB of Yonsei University Wonju Severance Christian Hospital (CR320318).

Patients were included if they underwent general anesthesia for diagnostic or surgical interventions. Cardiac, thoracic, and major vascular surgeries were excluded due to their distinct hemodynamic profiles, high use of invasive monitoring and vasoactive agents, and differing definitions and management strategies for IOH, which could introduce heterogeneity and limit model generalizability (See Fig A in S1 Appendix for the detailed patient flow). General anesthesia was defined as the administration of sedative agents combined with invasive mechanical ventilation via a laryngeal mask airway, endotracheal intubation, or tracheostomy. After applying the inclusion and exclusion criteria, a total of 319,699 cases were retained for model development and validation. This study followed the TRIPOD-AI (Transparent Reporting of a multivariable prediction model for Individual Prognosis Or Diagnosis—Artificial Intelligence) guidelines for developing and validating machine learning-based clinical prediction models [25]. The completed checklist is provided in the Supporting information (S1 Checklist), adapted from the TRIPOD Statement (https://www.tripod-statement.org/).

### Nested cohort study for clinical relevance

To evaluate the clinical impact of IOH, the nested cohort comprises adult inpatient surgical cases from the most recent full year (2023) in our institutional dataset, restricted to patients with at least one preoperative and one postoperative serum creatinine measurement. We excluded patients with pre-existing renal dysfunction (preoperative AKI with elevated baseline creatinine, established chronic kidney disease, end-stage renal disease on dialysis, or other conditions resulting in dialysis dependency).

Patients were stratified based on their cumulative exposure to intraoperative MAP thresholds of ≤65, ≤60, and ≤55 mmHg, measured in mmHg·minutes. The primary outcomes were the incidence of postoperative AKI within 7 days and AKD between 8 and 90 days after surgery, defined according to the Kidney Disease: Improving Global Outcomes (KDIGO) criteria and the Acute Disease Quality Initiative (ADQI) consensus definitions, respectively [26,27]. Covariates for multivariable adjustment were selected a priori using a directed acyclic graph (DAG) approach and include demographics (age, sex, BMI), preoperative comorbidities (hypertension, diabetes, coronary artery disease, baseline renal function), and intraoperative variables (surgery duration, fluid balance, vasopressor use, and estimated blood loss). We report the incidence of AKI/AKD and a full comparison of baseline and intraoperative characteristics between patients with and without AKI/AKD in Tables A and B in S1 Appendix.

### Definition of hypotension and data processing

In this study, MAP values were extracted directly from intraoperative bedside monitors using backend data interfaces. IOH was defined as a MAP <65 mmHg sustained for at least 1 min, based on established evidence linking this threshold to an increased risk of postoperative complications such as myocardial injury and acute kidney injury (AKI) [4,5,28].

Continuous vital sign data were acquired via network-based interfaces (e.g., TCP/IP) from standard clinical monitors. The extracted physiological parameters included heart rate, pulse rate, systolic and diastolic blood pressure, MAP, respiratory rate, peripheral oxygen saturation (SpO_2_), end-tidal carbon dioxide (ETCO_2_). All parameters were aligned to a uniform 1-min time grid to facilitate time-series analysis. All physiological variables were resampled to a uniform 1-min interval before model training. For parameters that were measured less frequently in the raw records (for example, non-invasive blood pressure), within-case forward-filling was applied until the next recorded value, consistent with clinical practice where a stable reading is assumed to persist until a new measurement is taken. Forward-fill was chosen over interpolation to reflect clinical practice, assuming stable values persist until the next measurement, while interpolation was applied only in sensitivity analyses to assess robustness. This preprocessing enabled consistent modeling while preserving the temporal structure of intraoperative physiological trends.

Prior to modeling, a comprehensive preprocessing pipeline was implemented to ensure data integrity and reliability. Physiologically implausible values were excluded based on predefined clinical thresholds (e.g., MAP <30 or >200 mmHg, HR <25 or >220 bpm, SpO_2_ <50% or >100%, ETCO_2_ <5 or >80 mmHg). Statistical outliers beyond the 0.1st and 99.9th percentiles of each parameter distribution were truncated to these limits. Cases with >10% missing vital sign data were excluded. Comparative analysis showed that cases excluded due to >10% missing data or physiologically implausible values did not materially alter the overall distributions of heart rate, MAP, or SpO_2_, supporting representativeness of the retained cohort. For the remaining data, missing values were imputed using physiological substitution formulas (e.g., MAP = [⅓ × DBP + ⅔ × SBP]) or forward-filling within the same case to preserve temporal continuity. Following cleaning, less than 0.5% of all timepoints were removed or truncated. Detailed missing and abnormal value statistics for both the internal and external datasets are presented in Tables C and D in S1 Appendix. The full dataset was then randomly partitioned into training (80%) and validation (20%) cohorts, with patient IDs used to ensure non-overlapping allocation across groups. The dataset of 319,699 cases was partitioned by patient ID to prevent data leakage at the patient level. Patients who underwent multiple surgical procedures were treated as separate cases, with each surgery considered an independent case. Such repeated surgeries accounted for approximately 5% of the total dataset.

### Model development and validation

We developed a deep learning model, termed HPI_Transformer (Fig B in S1 Appendix), based on the standard Transformer architecture, to enable real-time prediction of IOH. Our approach employed a conventional Transformer structure optimized for analyzing time-series vital-sign data. Unlike prior Temporal Fusion Transformer (TFT)-based studies that incorporated both static and dynamic variables [29] (e.g., laboratory results or medication data), we focused exclusively on vital signs to enhance interpretability, reduce input feature complexity, and facilitate real-time clinical implementation [30–32].

The model was trained to predict the onset of IOH at three clinically relevant future time points: 5, 10, and 15 min prior to the event. Input data consisted of continuous intraoperative physiological measurements, including heart rate, blood pressure (systolic, diastolic, and MAP), SpO_2_, respiratory rate, and ETCO_2_. Separate Transformer models were trained for each prediction horizon (5, 10, and 15 min). For each model, positive labels were assigned to 1-min epochs preceding an IOH event (MAP < 65 mmHg) occurring within the corresponding future interval, while negative labels were derived from epochs without hypotension in that window. The model architecture and hyperparameters were identical across horizons, but each model was trained on an independently constructed dataset tailored to its prediction interval.

To extract meaningful temporal features, a one-dimensional convolutional layer (Conv1D) was first applied to capture short-term patterns. Positional encoding was then incorporated to preserve the sequential order of the time series. The encoded sequences were passed through a Transformer encoder comprising multi-head self-attention layers, position-wise feedforward networks, and layer normalization, allowing the model to learn long-range temporal dependencies. A global average pooling layer aggregated the latent representations, which were then fed into a classification head with a sigmoid activation to output the probability of IOH.

Model input consisted of the 3 most recent time points (i.e., 5-, 10-, and 15-min prediction horizons) to forecast IOH risk across all prediction horizons. Cases lacking sufficient surgical duration to accommodate both the input and prediction windows were excluded. The model was evaluated at 5-, 10-, and 15-min prediction horizons, and performance remained stable across these intervals, demonstrating its adaptability for different real-time clinical use cases.

Hyperparameters—including learning rate, batch size, number of attention heads, hidden units, dropout rate, and input sequence length—were optimized using random search. Model training was guided by continuous monitoring of validation loss every 10 epochs, with early stopping applied after three consecutive epochs without improvement. Three independent Transformer models were trained for 5-, 10-, and 15-min prediction horizons, sharing an identical architecture and training configuration. Each model consisted of an 8-layer Transformer encoder with 8 attention heads, a model dimension (*d*_model_) of 64, and a feed-forward dimension of 256. Input data included 17 dynamic and 2 static features with a sequence length of 15 timepoints (1-min intervals). The models were trained for 200 epochs using the AdamW optimizer (learning rate = 2 × 10^−4^, batch size = 400–1,000) and a composite loss combining Focal Loss (*α* = 0.6, *γ* = 2) and an AUC-ranking term to address class imbalance. Dropout was set to 0.1 to mitigate overfitting. Hyperparameter selection was performed through grid search and empirical validation using the internal dataset. The same architecture was applied to all three prediction horizons to ensure comparability of results. Full hyperparameter settings and training configurations are provided in Fig B and Table E in S1 Appendix.

Model performance was evaluated through both internal and external validation. The internal validation set comprised a 20% holdout of the original dataset. For external validation, we used the Vital Signs DataBase (VitalDB) [33], which contains high-resolution intraoperative data from 6,388 patients. The external VitalDB dataset was preprocessed and standardized to match the internal data structure, including variable alignment, 1-min temporal resampling, outlier handling, and z-score normalization. To maintain consistency with the internal cohort, cardiac, thoracic, and major vascular surgeries were excluded. Detailed preprocessing procedures are described in Method A in S1 Appendix.

To benchmark performance, XGBoost models were trained using the same dynamic vital sign inputs and derived physiological and temporal features optimized for tree-based learning (Method B in S1 Appendix). Three separate models (5-, 10-, and 15-min prediction horizons) were developed with identical hyperparameter tuning procedures using grid search and 5-fold cross-validation.

Both models were implemented with practical computational efficiency in mind. The Transformer models were trained on a GPU workstation (NVIDIA GeForce RTX 3080, 10 GB VRAM) requiring approximately 8–12 GB of memory and <100 ms per prediction during inference, whereas the XGBoost models were trained on CPU hardware (Apple M2 Ultra) requiring ~8 GB memory and <10 ms per prediction. Full details of computational requirements are provided in Table F in S1 Appendix.

### Model evaluation

To comprehensively assess model performance, we employed multiple evaluation metrics covering discrimination ability, classification accuracy, and calibration reliability. Discrimination was quantified using receiver operating characteristic (ROC) curves and area under the ROC curve (AUC-ROC, 95% confidence interval [CI]), reflecting the model’s ability to distinguish hypotensive from non-hypotensive cases. Given the low prevalence of IOH, we further examined precision-recall (PR) curves and AUC-PR, which offer more reliable insights for imbalanced datasets. Classification accuracy was analyzed alongside F1 score, ensuring a balanced evaluation of precision and recall, while specificity was included to measure the model’s effectiveness in identifying non-hypotensive patients. Finally, calibration curves and expected calibration error (ECE, 95% CI) were used to determine the alignment between predicted probabilities and observed IOH incidence, ensuring robust risk estimation. These complementary metrics provided a multi-dimensional framework for evaluating predictive performance, supporting the clinical applicability of the model in real-time intraoperative monitoring.

### Simulation of the alert system

To assess the real-time feasibility and clinical interpretability of the model, we performed an alert simulation using 10 representative surgical cases randomly selected from the internal cohort. All selected cases had complete continuous intraoperative vital sign recordings and varied in operative duration to capture heterogeneous hemodynamic patterns. Using the trained Transformer model, IOH risk probabilities for MAP <65 mmHg were generated at 1-min intervals over the course of each procedure. Predicted risk trajectories were aligned with the corresponding minute-level MAP measurements to enable direct temporal comparison. An alert was considered triggered whenever the predicted probability exceeded the predefined decision threshold derived from the internal validation set. This simulation was designed to qualitatively evaluate temporal concordance between predicted IOH risk and observed hypotensive events, as well as the practical alert frequency during continuous monitoring, rather than to provide formal performance estimates.

### Sensitivity analysis and subgroup analyses

To evaluate model robustness and generalisability, several pre-specified sensitivity analyses were conducted. First, the predictive models were tested in high-risk surgical subgroups that were excluded from the primary cohort, including cardiac (*n* = 23,629), thoracic (*n* = 8,164), and vascular surgeries (*n* = 12,970), using identical preprocessing and feature engineering pipelines. Second, alternative definitions of IOH (MAP < 60 mmHg and <55 mmHg) were applied in both the internal and external validation cohorts to examine stability across different clinical thresholds. Third, to assess potential bias introduced by non-invasive blood pressure resampling, linear interpolation was applied prior to 1-min aggregation and model performance was re-evaluated.

Further subgroup analyses stratified by age (<65 versus ≥65 years) and American Society of Anesthesiologists (ASA) physical status (≤II versus ≥III) were performed to investigate performance variation across demographic and risk profiles. Consistency of discrimination, operating characteristics, and calibration was examined and summarized across all sensitivity and subgroup evaluations.

### Statistical analysis

Formal sample size calculation was not performed due to the retrospective nature and large scale of the dataset. For model development, the inclusion of 319,699 surgical cases provided ample statistical power to support training of high-capacity deep learning models and to ensure robust generalization. Due to privacy regulations and institutional policies, training datasets cannot be publicly released. However, external validation datasets from VitalDB are openly available, enabling replication of validation procedures (https://vitaldb.net). The code used for model training and evaluation is publicly available at https://github.com/ShouqiangZhu/IOH_Transformer and archived on Zenodo (version v1.0, https://zenodo.org/records/18932576).

All computations were performed using R 4.4.1, Python 3.12, and CUDA 12.2. Odds ratios (ORs) and 95% CIs for AKI and AKD were calculated per 60 mmHg·min increment of hypotensive exposure, using multivariable logistic regression models adjusted for baseline demographics, comorbidities, surgical type, and intraoperative confounders.

## Results

### Baseline characteristics

**Fig 1:**
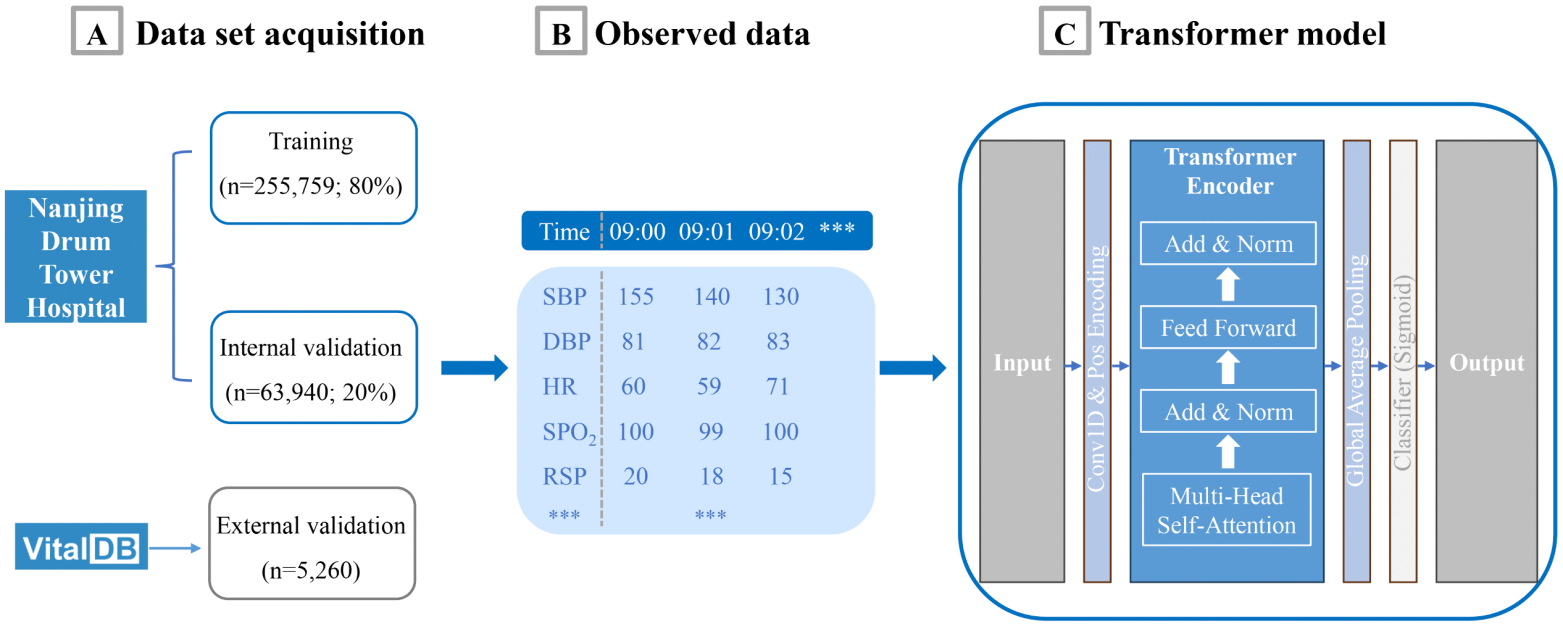
Overview of data acquisition, observed vital signs, and Transformer model architecture. **(A)** Data Acquisition: Patient data was obtained from Nanjing Drum Tower Hospital and VitalDB. The dataset from Nanjing Drum Tower Hospital was split into a training set (*n* = 255,759; 80%) and an internal validation set (*n* = 63,940; 20%), while external validation was performed using VitalDB (*n* = 5,260). **(B)** Observed Vital Signs: 12 physiological parameters were monitored in this study, including, body temperature, heart rate (HR), mean arterial pressure (MAP), end-tidal carbon dioxide (ETCO_2_), pulse rate, respiratory rate (RSP), blood oxygen saturation (SpO_2_), diastolic blood pressure (DBP), systolic blood pressure (SBP), non-invasive systolic blood pressure and non-invasive diastolic blood pressure. These variables were recorded at sequential time intervals. **(C)** Transformer Model Architecture: The physiological data were analyzed using a Transformer-based deep learning model, which incorporated key components such as self-attention mechanisms, feed-forward layers, and layer normalization to enable real-time intraoperative hypotension prediction.

A total of 319,699 patients were included in the study (Table 1 and Fig 1). The median age was 54 years (interquartile range [IQR], 40–65), and 48.73% (*n* = 155,789) of patients were male. The distribution of ASA physical status classifications showed that 56.33% of patients were classified as ASA ≥III (*n* = 180,093), while 43.67% were classified as ASA I/II (*n* = 139,606). Elective procedures constituted the majority of surgeries (92.26%), with emergency or urgent procedures accounting for 7.74%. The median surgical duration was 100.8 min (IQR, 57.6–158.4). Surgical disciplines were diverse, with the highest representation from gastrointestinal/colorectal surgery (21.77%), orthopedics (20.82%), and urology (14.62%).

**Table 1:** Baseline characteristics.

Variables	*N* = 319,699
Age (years)	54 (40–65)
Male sex	155,789 (48.73%)
ASA score	
I/II	139,606 (43.67%)
≥III	180,093 (56.33%)
Surgical urgency	
Elective	294,943 (92.26%)
Emergency/Urgent	24,756 (7.74%)
Duration of surgery (min)	100.8 (57.6-158.4)
Surgical discipline	
Gastrointestinal/Colorectal Surgery	69,587 (21.77%)
Orthopedics	66,559 (20.82%)
Urology	46,751 (14.62%)
General surgery	34,699 (10.85%)
Gynecology/Obstetrics	27,109 (8.48%)
Hepatobiliary Surgery	23,708 (7.42%)
Neurosurgery	22,448 (7.02%)
ENT	17,823 (5.57%)
Pancreatic and Metabolic Surgery	5,146 (1.61%)
Burn Plastic Surgery	3,667 (1.15%)
Other	2,202 (0.69%)

Data were collected from adult patients undergoing various elective and emergency surgeries at a tertiary hospital in China between 2013 and 2023. Values are presented as median (interquartile range) for continuous variables and number (percentage) for categorical variables. ASA, American Society of Anesthesiologists physical status; ENT, Ear, Nose, and Throat.

### Association of IOH burden with AKI and AKD

**Fig 2:**
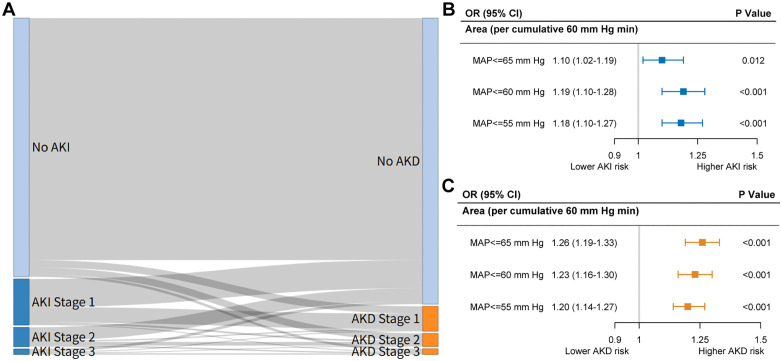
Association between intraoperative hypotension burden and postoperative kidney injury risk. The left panel illustrates the transitions in postoperative kidney status using a Sankey diagram, showing the flow of patients from no injury to different stages of acute kidney injury and acute kidney disease **(A)**. The upper-right forest plot presents the association between cumulative hypotension burden (per 60 mmHg·min below MAP thresholds) and the risk of AKI **(B)**, while the lower-right plot shows corresponding risks for AKD **(C)**. Odds ratios and 95% confidence intervals were estimated using logistic regression models, and corresponding P values were derived from these models. Abbreviations: AKI, acute kidney injury; AKD, acute kidney disease; MAP, mean arterial pressure.

A nested cohort analysis was performed to evaluate the association between IOH burden and postoperative kidney complications. Among 23,075 patients with available perioperative serum creatinine data, 3,610 (15.6%) developed postoperative AKI within 7 days after surgery. For the assessment of acute kidney disease (AKD), which requires serum creatinine measurement between postoperative days 8 and 90, 1,139 patients (4.9%) met the diagnostic criteria. Baseline characteristics stratified by AKI and AKD status are presented in Tables A and B in S1 Appendix. In the nested cohort analysis, cumulative exposure to IOHwas significantly associated with increased postoperative renal risk (Fig 2). Each additional 60 mmHg·min of MAP ≤65 mmHg was associated with an adjusted OR of 1.10 (95% CI, [1.02, 1.19]; *p* = 0.012) for postoperative AKI and 1.26 (95% CI, [1.19, 1.33]; *p* < 0.001) for AKD. Similar trends were observed at MAP thresholds of ≤60 mmHg (AKI OR: 1.19; AKD OR: 1.23) and ≤55 mmHg (AKI OR: 1.18; AKD OR: 1.20), demonstrating a consistent and incremental association between hypotension burden and renal complications. A Sankey diagram (Fig 2) illustrates the progression from AKI to AKD. Among patients with postoperative AKI, a substantial proportion progressed to AKD, with increasing transition rates observed at higher AKI stages. Notably, most patients without AKI did not develop AKD, while those with AKI Stage 2 or 3 showed prominent flows into AKD Stage 2 or 3, highlighting the continuum of renal injury beyond the immediate postoperative period.

### Model performance for hypotension prediction

**Fig 3:**
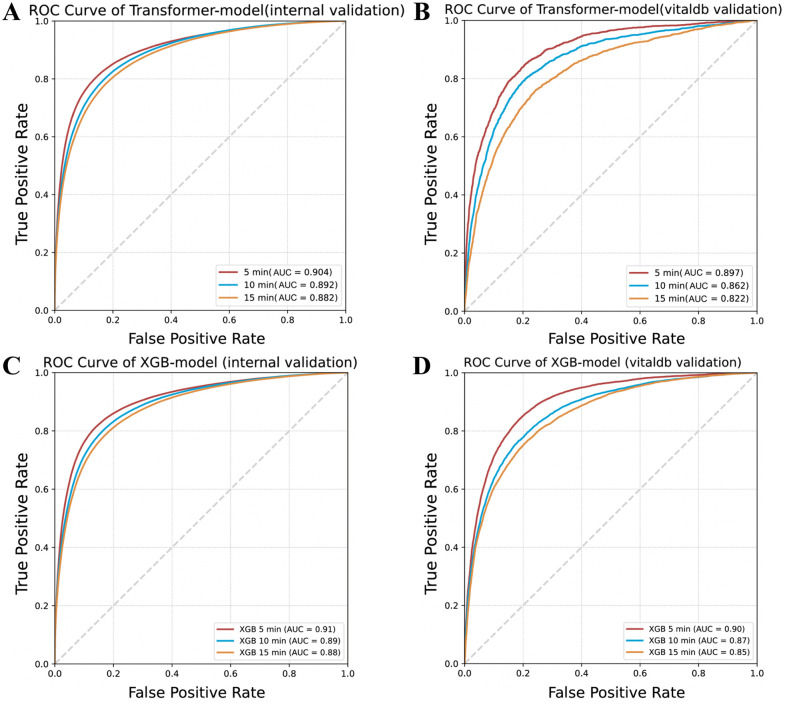
Comparative model performance for real-time prediction of intraoperative hypotension. Receiver operating characteristic (ROC) curves comparing the predictive performance of the Transformer-based and XGBoost-based models for intraoperative hypotension across multiple time horizons. Both models were validated using internal data from Nanjing Drum Tower Hospital and external data from VitalDB. The curves illustrate model discrimination ability at 5-, 10-, and 15-min prediction windows, with respective area under the curve (AUC) values indicated for each validation cohort. Higher AUC values represent improved hypotension detection accuracy.

**Fig 4:**
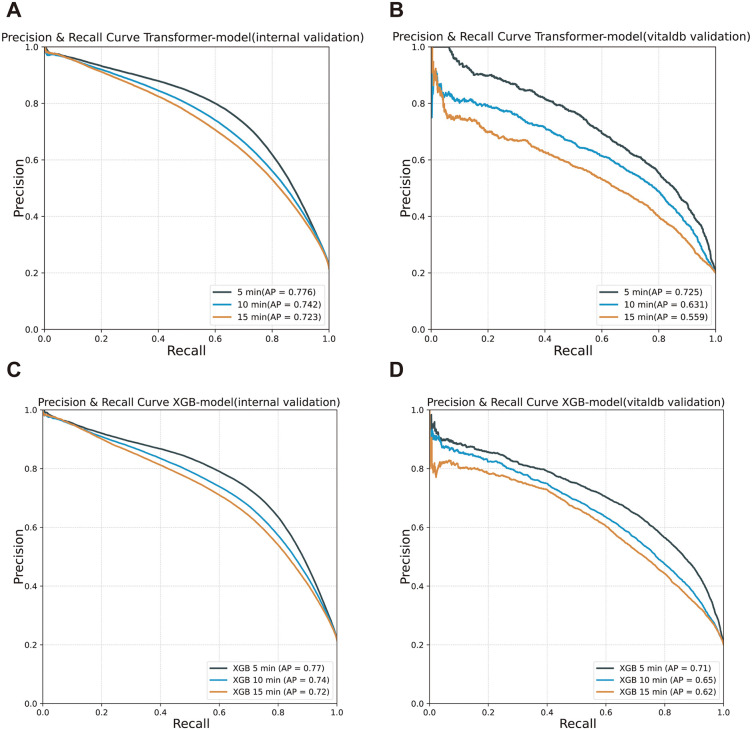
Precision-recall (PR) curves comparing Transformer-based and XGB models for intraoperative hypotension prediction. Precision-recall curves illustrating the predictive performance of the Transformer-based and XGB-based models across internal and external validation datasets. Each plot represents model performance at different time intervals (5, 10, and 15 min) for hypotension prediction. The Average Precision (AP) scores indicate overall classification effectiveness, with higher AP values reflecting better precision-recall balance.

The Transformer-based model demonstrated strong predictive performance for IOH (Table 2, Figs 3 and 4). In internal validation, it achieved an AUC of 0.904 at 5 min, 0.892 at 10 min, and 0.882 at 15 min, with a recall of 89.1%, 88.3%, and 89.6%, respectively. The external validation results showed slight decreases in AUC (0.897, 0.862, and 0.822 at 5, 10, and 15 min, respectively) but retained high recall values. In contrast, the XGB-based model demonstrated higher accuracy and specificity, achieving an AUC of 0.905 at 5 min, 0.892 at 10 min, and 0.882 at 15 min, suggesting a more conservative detection approach. In comparative analyses (Tables 2 and 3), the Transformer and XGBoost models demonstrated complementary strengths, with trade-offs that depended on validation cohort and performance metric. In internal validation at the 5-min horizon, the Transformer achieved higher recall (0.891 versus 0.737) and substantially better probability calibration (ECE 0.0083 versus 0.0373) than XGBoost, whereas XGBoost showed higher overall accuracy (0.875 versus 0.759) and specificity (0.913 versus 0.723). Similar trade-offs between sensitivity and specificity were observed across longer prediction horizons. In external validation, the Transformer consistently maintained higher recall across all horizons, while calibration performance between the two models was comparable and varied by forecast window. Across cohorts, XGBoost generally favored higher precision and specificity, whereas the Transformer emphasized sensitivity and stable probabilistic outputs, particularly in the internal dataset.

**Table 2:** Performance comparison of Transformer-based and XGBoost-based models for real-time prediction of intraoperative hypotension at multiple time horizons.

Models	Accuracy	F1 score	PPV	Recall	Specificity	AUC (95% CI)
**Transformer-based model**						
Internal validation (5 min)	0.759	0.617	0.472	0.891	0.723	0.904 (0.903, 0.904)
Internal validation (10 min)	0.744	0.598	0.452	0.883	0.706	0.892 (0.891, 0.893)
Internal validation (15 min)	0.701	0.563	0.410	0.896	0.647	0.882 (0.881, 0.883)
VitalDB validation (5 min)	0.624	0.505	0.343	0.957	0.541	0.897 (0.890, 0.904)
VitalDB validation (10 min)	0.604	0.485	0.328	0.934	0.521	0.862 (0.853, 0.870)
VitalDB validation (15 min)	0.592	0.467	0.316	0.895	0.516	0.822 (0.812, 0.832)
**XGB-based model**						
Internal validation (5 min)	0.875	0.719	0.702	0.737	0.913	0.905 (0.905, 0.906)
Internal validation (10 min)	0.864	0.686	0.679	0.693	0.910	0.892 (0.891, 0.892)
Internal validation (15 min)	0.856	0.667	0.665	0.668	0.908	0.882 (0.881, 0.882)
VitalDB validation (5 min)	0.752	0.595	0.442	0.912	0.712	0.901 (0.897, 0.904)
VitalDB validation (10 min)	0.711	0.549	0.399	0.880	0.669	0.868 (0.865, 0.869)
VitalDB validation (15 min)	0.692	0.529	0.381	0.863	0.650	0.853 (0.852, 0.856)

Abbreviations: PPV, positive predictive value; AUC, area under the receiver operating characteristic curve; CI, confidence interval; XGB, extreme gradient boosting. F1 score, harmonic mean of precision and recall.

**Table 3:** Comparison of expected calibration error between internal and external cohorts.

Forecast time	Internal validation		External validation	
	Transformer	XGB	Transformer	XGB
5 min	0.0083 (0.0077, 0.0089)	0.0373 (0.0369, 0.0376)	0.007 (0.0056, 0.0141)	0.0076 (0.0073, 0.0083)
10 min	0.011 (0.0104, 0.0117)	0.0334 (0.0331, 0.0336)	0.0059 (0.0051, 0.0141)	0.0044 (0.0045, 0.0053)
15 min	0.0125 (0.0118, 0.0131)	0.0315 (0.0315, 0.0318)	0.0092 (0.0079, 0.0178)	0.0040 (0.0041, 0.0051)

### Calibration analysis

**Fig 5:**
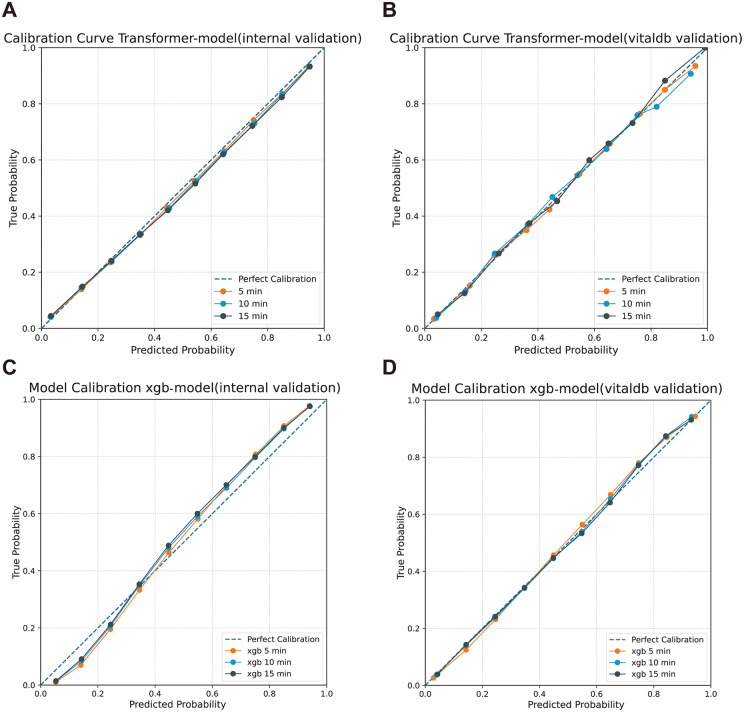
Calibration curves comparing Transformer-based and XGB-based models for intraoperative hypotension prediction. The calibration curves illustrate the agreement between predicted probabilities and true probabilities for intraoperative hypotension across different models and validation settings. Each plot displays calibration performance at 5-, 10-, and 15-min prediction horizons, with a dashed line representing perfect calibration for reference. **(A)** Transformer Model (Internal Validation): Calibration performance using patient data from Nanjing Drum Tower Hospital. **(B)** Transformer Model (External Validation—VitalDB): Model generalizability assessment with independent VitalDB dataset. **(C)** XGB Model (Internal Validation): Calibration results for the XGB model on the internal validation dataset. **(D)** XGB Model (External Validation—VitalDB): XGB model calibration evaluation using external validation data from VitalDB.

Calibration curves (Fig 5) indicated that the Transformer model exhibited better alignment between predicted and actual probabilities across time horizons, particularly in external validation, where calibration errors remained minimal (Table 3). At 5 min, the ECE for the Transformer model was 0.007, compared to 0.0076 for XGB. At 10 and 15 min, the Transformer model exhibited lower ECE than XGB in the internal validation cohort, whereas both models demonstrated low ECE values in the external validation cohort, with XGB showing slightly lower calibration error at longer prediction horizons.

### Alert system simulation results

To illustrate the clinical interpretability of the Transformer model, trajectory analyses were performed on 10 representative surgeries randomly selected from the internal validation cohort (total duration = 1,221 min; mean ± SD = 122.1 ± 40.2 min per case). Across these cases, 87 actual IOH events occurred (mean 8.7 per surgery). Fig C in S1 Appendix presents a representative case, showing coherent temporal alignment between predicted IOH probabilities at 5-, 10-, and 15-min horizons and observed MAP fluctuations, illustrating the model’s capacity for real-time IOH alerting.

### Additional sensitivity analysis and subgroup analyses

In addition to the primary analyses, multiple sensitivity analyses were conducted to evaluate model robustness and generalisability. First, among high-risk cardiac, thoracic, and vascular subgroups, the Transformer model maintained AUCs >0.87 across all horizons, confirming robust generalizability (Table G in S1 Appendix). Second, alternative IOH definitions (MAP <60 mmHg and <55 mmHg) were applied. Model performance remained stable across thresholds (Tables H and I in S1 Appendix), supporting the robustness of the predictive task independent of a specific cutoff. Third, we evaluated the impact of non-invasive blood pressure processing by applying linear interpolation prior to resampling, yielding comparable performance, indicating minimal influence of temporal alignment methodology (Table J in S1 Appendix). Finally, subgroup analyses across age (<65 versus ≥65 years) and ASA classification (≤II versus ≥III) showed minimal variation in key performance metrics for both Transformer and XGBoost models in internal and external cohorts, demonstrating strong generalisability across demographic and clinical risk strata (Tables K and L in S1 Appendix).

## Discussion

In this study, we developed and validated a Transformer-based deep learning model to predict IOH in real time, using only continuous vital sign time-series data, without relying on high-resolution waveforms or additional clinical inputs. Trained on a large-scale surgical cohort from a tertiary hospital and externally validated on an independent dataset, the model demonstrated strong and consistent predictive performance across multiple time horizons. To enhance interpretability and clinical applicability, we further conducted a trajectory analysis using 10 representative surgical cases from the internal validation cohort. In these examples, the predicted IOH risk probabilities dynamically tracked the temporal evolution of MAP and accurately anticipated hypotensive episodes, illustrating the model’s potential for real-time clinical monitoring. The predicted IOH trajectories in individual cases informed the design of our ongoing prospective randomized trial, guiding the timing of alert interventions to mitigate hypotensive burden in real time. Additionally, in our nested cohort analysis, we observed a consistent association between the burden of IOH and postoperative kidney complications, including AKI and AKD. While this relationship has been well established in previous large-scale studies, our findings reinforce its clinical importance and demonstrate how real-time IOH prediction could facilitate early intraoperative management to potentially mitigate renal risk. Together, these findings suggest that a vital sign–only, Transformer-based approach can provide an accurate, generalizable, and actionable solution for perioperative hemodynamic management.

While it is true that key intraoperative factors—such as anesthetic dosing, fluid administration, blood loss, surgical complexity, baseline blood pressure, and comorbidities—strongly influence IOH, existing models must balance accuracy with practicality. Wijnberge and colleagues note that vital-signs–only models “do not take factors into account such as the administration of relevant anesthetic drugs and the sometimes (imminent) influence of surgical manipulations” [34]. Lee and colleagues demonstrated an AUC of 0.74 in a model incorporating rich clinical inputs, while deep-learning models relying solely on vital signs (e.g., Transformer-based) have achieved AUCs >0.90 in large datasets [19]. Moreover, in ICU settings, models using only vital signs have reported AUCs of 0.93 and 0.88 for hypotension events 15 and 60 min in advance [18], respectively, suggesting these signals can implicitly capture the effects of interventions and comorbidity interactions.

Our rationale for prioritizing a vital-signs-only approach stems from three considerations: 1) Scalability and Real-World Feasibility. In many low- and middle-income settings or non-research operation rooms, precise data on drug dosing, fluids, blood loss, or surgical complexity may not be available in machine-readable format. Our model intentionally uses only time-series vital signs—universally captured and immediately accessible—to ensure broad applicability. 2) Model Performance versus Complexity Trade-off. While incorporating additional variables can increase accuracy, the marginal gains may be limited. For example, a machine-learning model for post-induction hypotension achieved an AUROC of approximately 0.74 [35], whereas vital-sign models (including our Transformer) can reach comparable or higher discrimination in large datasets (e.g., AUC >0.90 for 5‑min IOH prediction). 3) Inter-variable Interactions Captured Implicitly. Even without explicit drug or fluid data, vital sign fluctuations often reflect the effects of these interventions: vasopressors raise BP, blood loss decreases it, et cetera. Transformer models, with their temporal self-attention mechanisms, are adept at modeling such dynamic interactions. As noted in physiological literature, BP dynamics arise from “heart rate × stroke volume × systemic vascular resistance,” illustrating how vital signs can proxy complex interventions and comorbidity effects.

To further address the debated clinical value of short-term IOH prediction, we incorporated a nested retrospective cohort analysis examining the association between cumulative hypotension burden and postoperative renal outcomes. We found that greater cumulative exposure to MAP below 65, 60, and 55 mmHg was independently associated with increased risks of both AKI and AKD, demonstrating a consistent and incremental association. These findings support the pathophysiologic relevance of IOH in non-cardiac surgical populations and affirm the importance of early recognition and intervention. Previous studies have raised questions about the causal role of IOH in postoperative kidney injury. In a large cohort of cardiac surgery patients, although IOH was associated with AKI, it was not linked to the development of AKD, where venous congestion (elevated CVP) appeared to be the dominant risk factor—likely reflecting the distinct hemodynamic physiology of cardiopulmonary bypass [36]. Additionally, A recent multicenter randomized trial involving 917 moderate- to high-risk elective abdominal surgery patients found that HPI-guided hemodynamic management significantly reduced the duration of hypotensive episodes (TWA‑MAP <65 mmHg), but did not meaningfully decrease the incidence of moderate-to-severe AKI (6.1% versus 7.0%, RR 0.89, 95% CI 0.54, 1.49; *P* = 0.66) [37]. While this suggests HPI can effectively monitor and limit IOH, the lack of benefit in AKI reduction may reflect study-specific factors—such as limited intervention follow-through, patient selection nuances, or sample size—as has been noted in other trials. In contrast, our study analyzes a large-scale, heterogeneous non-cardiac surgical cohort (319,699 cases) that includes but is not limited to abdominal procedures, providing broader applicability than prior abdominal-only RCTs. We demonstrate a consistent and incremental association between cumulative hypotension burden and both postoperative AKI and AKD after multivariable adjustment using clinically informed covariates. This suggests that, while isolated use of hypotension alerts may not always translate into improved outcomes, targeting IOH burden as a modifiable risk factor across diverse surgical populations remains clinically meaningful. Accordingly, our Transformer-based model provides a framework for reliable short-term IOH risk prediction and may support the development of clinically informed alerting strategies in real-world perioperative settings.

A key strength of our model is its ability to integrate routinely monitored vital sign parameters, enhancing its utility across diverse surgical settings with varying monitoring complexities. In contrast to traditional approaches such as the HPI, which relies solely on arterial waveform analysis [14], our model utilizes routine vital signs, minimizing the dependence on specialized monitoring equipment. This feature makes the model highly adaptable and accessible, potentially improving perioperative care in a wide range of clinical environments. Furthermore, the Transformer model demonstrated performance comparable to that of conventional machine learning algorithms, while offering a flexible framework for modeling temporal dependencies within intraoperative time-series data, which is relevant for early IOH prediction [22,38].

Our comparison between the Transformer model and the XGBoost-based (XGB) model revealed distinct strengths and limitations. The Transformer model consistently exhibited higher recall, achieving a sensitivity of 89.1% at 5 min, 88.3% at 10 min, and 89.6% at 15 min, underscoring its capacity to detect IOH risk before clinical symptoms manifest. Both models were implemented with practical computational efficiency in mind. As expected, the XGBoost models achieved faster per-prediction inference (<10 ms) on CPU hardware, whereas the Transformer models required <100 ms per prediction. Importantly, the intended operational distinction between the two approaches does not lie in inference speed superiority. Rather, the Transformer architecture offers an alternative deployment paradigm by directly ingesting short fixed-length physiological time windows and modeling temporal dependencies end-to-end, without reliance on manually engineered temporal summary features. In contrast, tree-based models depend on explicitly constructed dynamic features that must be recomputed as new data arrive, increasing preprocessing complexity at the system level. These differences primarily affect pipeline design and integration with continuous monitoring systems, rather than discrimination performance or raw inference latency.

While our Transformer-based models demonstrated strong predictive performance, particularly in terms of sensitivity and probability calibration, the XGBoost models achieved similar or higher discrimination in some settings and consistently higher specificity and positive predictive value. These findings indicate a trade-off between early detection (higher recall) and minimizing false alerts (higher specificity). In clinical practice, higher recall may be preferred when the priority is early intervention to prevent end-organ injury, accepting a higher rate of false positives; conversely, higher specificity may be desirable in environments where alert fatigue is a dominant concern. Importantly, superior calibration—observed with the Transformer models—facilitates more reliable risk scoring and threshold selection. Therefore, we recommend that institutions considering deployment evaluate both model families, perform local calibration, and select operating points (or ensemble/stacking strategies) aligned with local workflows. Prospective, real-time implementation trials are necessary to determine which configuration yields the greatest patient benefit. While advanced variants such as the TFT or Informer may enhance long-horizon forecasting, they impose substantially higher compute costs and may be less suitable for real-time deployment in the operating room. Future work will explore these extended architectures as optimization in clinical hardware environments evolves. In practical deployment, the Transformer model may be preferred when early detection and high recall are prioritized, whereas XGBoost could be favored in settings where high specificity and reduced false alarms are critical. Notably, predictive performance slightly declined at 15-min horizons, suggesting that short-term forecasts may offer more reliable clinical guidance.

An additional observation is that ECE values were lower in the external VitalDB validation than in the internal cohort for both models, which may appear counterintuitive. This likely reflects differences in cohort characteristics, including standardized data acquisition and reduced signal noise in the external dataset, which can improve the alignment between predicted probabilities and observed outcomes. Differences in event prevalence and risk distributions may also result in more conservative probability estimates, thereby reducing calibration error without necessarily improving discrimination performance. Similar findings regarding external calibration improvement have been reported in prior studies of machine learning models applied to heterogeneous clinical datasets [39,40]

Subgroup analyses showed consistent model performance across age and ASA-based risk strata in both development and external cohorts, suggesting the approach generalizes well across diverse surgical populations. These findings, combined with robustness to measurement-processing variations and multiple IOH definitions, further support the clinical transferability of the prediction models.

Our study has several limitations. First, it is a retrospective analysis, and prospective validation in diverse clinical settings is required to confirm the real-time applicability of our model. Although we validated our findings with external data from VitalDB, multicenter studies across a wider variety of surgical populations are necessary to evaluate the reproducibility and generalizability of our results. Second, while the Transformer model demonstrated superior recall, optimizing both sensitivity and specificity for different patient subgroups remains a challenge. Future studies should consider hybrid modeling approaches that combine the sequential processing strengths of Transformer with the structured feature importance of XGB to enhance prediction accuracy. Thirdly, the reliance on real-time patient monitoring and integration with electronic health records (EHRs) may limit the model’s applicability in settings without seamless EHR integration, a challenge that should be addressed in future work. Finally, because this study is retrospective, causal inference between model use and postoperative outcomes cannot be established. We acknowledge that demonstrating a causal pathway from model-guided hemodynamic management to reduced IOH burden and fewer postoperative complications requires prospective interventional validation. A prospective randomized controlled trial initiated by our research team has completed institutional ethical defense and is currently under formal ethics review. This ongoing study aims to evaluate whether real-time deployment of our prediction model can effectively reduce the incidence of intraoperative hypotension and mitigate postoperative renal and other organ-specific complications.

## Supporting information

S1 Appendix **Table A.** Comparison of baseline characteristics between AKI patients and control group. **Table B.** Comparison of baseline characteristics between AKD patients and control group. **Table C.** Summary of data completeness and outlier detection for the internal dataset (Nanjing Drum Tower Hospital, 2013–2023). **Table D.** Summary of data completeness and outlier detection for the external validation dataset (VitalDB, South Korea). **Table E.** Transformer model architecture, hyperparameters, and training configuration. **Table F.** Computational requirements for Transformer and XGBoost models. **Table G.** Performance of the XGBoost and Transformer model in high-risk surgical subgroups (cardiac, thoracic, and vascular surgeries). **Table H.** Sensitivity analysis of XGBoost and Transformer models under an alternative IOH definition (MAP < 55 mmHg). **Table I.** Sensitivity analysis of XGBoost and Transformer models under an alternative IOH definition (MAP < 60 mmHg). **Table J.** Performance of XGBoost and Transformer models after interpolation-based preprocessing of non-invasive blood pressure measurements for 5-min IOH prediction in internal and external validation datasets. **Table K.** Subgroup performance of the XGBoost model for 5-min intraoperative hypotension prediction across age and ASA risk categories in internal and external validation cohorts. **Table L.** Subgroup performance of the Transformer model for 5-min intraoperative hypotension prediction across age and ASA risk categories in internal and external validation cohorts. **Fig A.** Flowchart showing patient selection for the model development cohort at Nanjing Drum Tower Hospital. **Fig B.** HPI Transformer architecture. Each of the three Transformer classifiers (5-, 10-, and 15-min prediction horizons) employed an 8-layer encoder with 8 attention heads, a model dimension (*d*_model_) of 64, feed-forward dimension 256, and dropout 0.1. Models were trained for 200 epochs using the AdamW optimizer (learning rate = 2 × 10⁻⁴, batch size = 400–1,000) with a combined Focal Loss (*α* = 0.6, *γ* = 2) and AUC-ranking loss. Full details, including feature definitions, tuning parameters, and hardware configuration, are provided in Table 5 in S1 Appendix. **Fig C.** Intraoperative mean arterial pressure (MAP) trajectories and Transformer-based predictions of intraoperative hypotension (IOH) risk in a representative surgical case. The top panel displays the intraoperative mean arterial pressure (MAP) trajectory, where the solid blue line represents continuous MAP measurements. Orange circles indicate normotensive states (MAP ≥ 65 mmHg), while red circles denote hypotensive episodes (MAP < 65 mmHg). The black dashed horizontal line marks the clinical hypotension threshold at 65 mmHg, with shaded backgrounds highlighting normotensive (light orange) and hypotensive (light red) ranges. The lower panels show Transformer-based predicted intraoperative hypotension (IOH) risk probabilities at 5-, 10-, and 15-min prediction horizons, represented by blue, orange, and purple lines, respectively. Red dashed lines in each prediction panel indicate the predefined risk threshold. Insets summarize the mean and maximum predicted probabilities, as well as the total number of predicted IOH events exceeding the threshold. **Method A.** Preprocessing and standardization of the VitalDB dataset. **Methods B.** XGBoost model configuration and hyperparameter tuning.
(PDF)

S1 Study Protocol Study protocol for the development and external validation of a Transformer-based model for real-time prediction of intraoperative hypotension using dynamic time-series vital signs. (DOC)

S1 Checklist TRIPOD-AI reporting checklist. Adapted from the TRIPOD Statement (Transparent Reporting of a multivariable prediction model for Individual Prognosis Or Diagnosis). Available from: https://www.tripod-statement.org/. TRIPOD Statement. Distributed under the Creative Commons Attribution 4.0 International License (CC BY 4.0).
(PDF)

## Abbreviations

ADQI: Acute Disease Quality Initiative
AKD: acute kidney disease
AKI: acute kidney injury
ASA: American Society of Anesthesiologists
CI: confidence interval
Conv1D: one-dimensional convolutional layer
DAG: directed acyclic graph
ECE: expected calibration error
EHRs: electronic health records
ETCO_2_: end-tidal carbon dioxide
HPI: Hypotension Prediction Index
IOH: intraoperative hypotension
IQR: interquartile range
KDIGO: Kidney Disease: Improving Global Outcomes
MAP: mean arterial pressure
ORs: odds ratios
PR: precision-recall
ROC: receiver operating characteristic
SpO_2_: peripheral oxygen saturation
TFT: Temporal Fusion Transformer
VitalDB: Vital Signs DataBase
