# Supplementary material for: Transformer-based deep learning model for real-time prediction of intraoperative hypotension using dynamic time-series vital signs: A retrospective study
Source: PLoS Med. 2026 Mar 25;23(3):e1005024. doi: 10.1371/journal.pmed.1005024 (PMC13046278; doi:10.1371/journal.pmed.1005024)
Supplement: S1 Appendix — Table A. Comparison of baseline characteristics between AKI patients and control group. Table B. Comparison of baseline characteristics between AKD patients and control group. Table C. Summary of data completeness and outlier detection for the internal dataset (Nanjing Drum Tower Hospital, 2013–2023). Table D. Summary of data completeness and outlier detection for the external validation dataset (VitalDB, South Korea). Table E. Transformer model architecture, hyperparameters, and training configuration. Table F. Computational requirements for Transformer and XGBoost models. Table G. Performance of the XGBoost and Transformer model in high-risk surgical subgroups (cardiac, thoracic, and vascular surgeries). Table H. Sensitivity analysis of XGBoost and Transformer models under an alternative IOH definition (MAP < 55 mmHg). Table I. Sensitivity analysis of XGBoost and Transformer models under an alternative IOH definition (MAP < 60 mmHg). Table J. Performance of XGBoost and Transformer models after interpolation-based preprocessing of non-invasive blood pressure measurements for 5-min IOH prediction in internal and external validation datasets. Table K. Subgroup performance of the XGBoost model for 5-min intraoperative hypotension prediction across age and ASA risk categories in internal and external validation cohorts. Table L. Subgroup performance of the Transformer model for 5-min intraoperative hypotension prediction across age and ASA risk categories in internal and external validation cohorts. Fig A. Flowchart showing patient selection for the model development cohort at Nanjing Drum Tower Hospital. Fig B. HPI Transformer architecture. Each of the three Transformer classifiers (5-, 10-, and 15-min prediction horizons) employed an 8-layer encoder with 8 attention heads, a model dimension (dmodel) of 64, feed-forward dimension 256, and dropout 0.1. Models were trained for 200 epochs using the AdamW optimizer (learning rate = 2 × 10⁻⁴, batch size = 400–1,000) with a [file pmed.1005024.s001.pdf]

S1 Appendix

Table A. Comparison of baseline characteristics between AKI patients and control group.

Table B. Comparison of baseline characteristics between AKD patients and control group.

Table C. Summary of Data Completeness and Outlier Detection for the Internal Dataset (Nanjing Drum Tower Hospital, 2013–2023).

Table D. Summary of Data Completeness and Outlier Detection for the External Validation Dataset (VitalDB, South Korea).

Table E. Transformer Model Architecture, Hyperparameters, and Training Configuration.

Table F. Computational Requirements for Transformer and XGBoost Models.

Table G. Performance of the XGBoost and Transformer Model in High-Risk Surgical Subgroups (Cardiac, Thoracic, and Vascular Surgeries).

Table H. Sensitivity Analysis of XGBoost and Transformer Models Under an Alternative IOH Definition (MAP < 55 mmHg).

Table I. Sensitivity Analysis of XGBoost and Transformer Models Under an Alternative IOH Definition (MAP < 60 mmHg).

Table J. Performance of XGBoost and Transformer models after interpolation-based preprocessing of non-invasive blood pressure measurements for 5-minute IOH prediction in internal and external validation datasets.

Table K. Subgroup Performance of the XGBoost Model for 5-Minute Intraoperative Hypotension Prediction Across Age and ASA Risk Categories in Internal and External Validation Cohorts.

Table L. Subgroup Performance of the Transformer Model for 5-Minute Intraoperative Hypotension Prediction Across Age and ASA Risk Categories in Internal and External Validation Cohorts.

Figure A. Flowchart showing patient selection for the model development cohort at Nanjing Drum Tower Hospital.

Figure B. HPI transformer architecture.

Figure C. Intraoperative mean arterial pressure (MAP) trajectories and Transformer-based predictions of intraoperative hypotension (IOH) risk in a representative surgical case.

Method A. Preprocessing and Standardization of the VitalDB Dataset.

Methods B. XGBoost Model Configuration and Hyperparameter Tuning.

Supplemental Tables

Table A. Comparison of baseline characteristics between AKI patients and control group.

**Variables**	**Total (n = 23075)**	**Non-AKI group (n = 19465)**	**AKI group (n = 3610)**	***P***
**Demographic Characteristic**				
Sex, n(%)				<.001
Male	13036 (56.49%)	10545 (54.17%)	2491 (69%)	
Female	10039 (43.51%)	8920 (45.83%)	1119 (31%)	
Age (Year)	60 (50, 69)	59 (49, 69)	66 (56, 73)	<.001
Height (cm)	165 (160, 170.5)	165.00 (160, 170)	168 (160, 172)	<.001
Weight (kg)	65.00 (57.50, 75)	65.00 (57.5, 75)	65.00 (57.5, 74.5)	0.238
BMI (kg/m^2)	23.94 (21.51, 26.56)	24.03 (21.63, 26.63)	23.45 (21.26, 26.05)	<.001
ASA classification, n (%)				<.001
I	107 (0.46%)	80 (0.41%)	27 (0.75%)	
II	3215 (13.93%)	2950 (15.16%)	265 (7.34%)	
III	15994 (69.31%)	13845 (71.13%)	2149 (59.53%)	
IV	3247 (14.07%)	2303 (11.83%)	944 (26.15%)	
V	512 (2.22%)	287 (1.47%)	225 (6.23%)	
**Preoperative Comorbidities**				
Hypertension, n(%)				<.001
No	15415 (66.80%)	13304 (68.35%)	2111 (58.48%)	
Yes	7660 (33.20%)	6161 (31.65%)	1499 (41.52%)	
Diabetes Mellitus, n(%)				<.001
No	20355 (88.21%)	17319 (88.98%)	3036 (84.10%)	
Yes	2720 (11.79%)	2146 (11.02%)	574 (15.90%)	
History of Malignancy, n(%)				<.001
No	17058 (73.92%)	14821 (76.14%)	2237 (61.97%)	
Yes	6017 (26.08%)	4644 (23.86%)	1373 (38.03%)	
History of Stroke, n(%)				<.001
No	21323 (92.41%)	18074 (92.85%)	3249 (90%)	
Yes	1752 (7.59%)	1391 (7.15%)	361 (10%)	
Coronary Artery Disease, n(%)				<.001
No	21999 (95.34%)	18634 (95.73%)	3365 (93.21%)	

Variables	Total (n = 23075)	Non-AKI group (n = 19465)	AKI group (n = 3610)	*P*
Yes	1076 (4.66%)	831 (4.27%)	245 (6.79%)	
Valvular Heart Disease, n(%)				0.176
No	22973 (99.56%)	19374 (99.53%)	3599 (99.70%)	
Yes	102 (0.44%)	91 (0.47%)	11 (0.30%)	
Liver Cirrhosis, n(%)				0.592
No	22832 (98.95%)	19257 (98.93%)	3575 (99.03%)	
Yes	243 (1.05%)	208 (1.07%)	35 (0.97%)	
Anemia, n(%)				<.001
No	22599 (97.94%)	19115 (98.20%)	3484 (96.51%)	
Yes	476 (2.06%)	350 (1.80%)	126 (3.49%)	
**Preoperative Laboratory Tests**				
Albumin (g/L)	39.90 (37.8, 41.9)	40.1 (38.10, 42)	38.80 (35.8, 41)	<.001
ALT (U/L)	17.30 (12.2, 26.70)	17.40 (12.4, 27)	17.00 (11.7, 25.5)	<.001
Creatinine (μmol/L)	62 (5, 74)	61.00 (51, 73)	68.00 (56, 85)	<.001
Direct Bilirubin (μmol/L)	2.10 (1.50, 3.10)	2.10 (1.50, 3.00)	2.40 (1.60, 3.70)	<.001
Glucose (mmol/L)	4.80 (4.37, 5.58)	4.77 (4.36, 5.48)	5.00 (4.46, 6.30)	<.001
Platelet Count ($\times 10^9$/L)	200 (159, 244.5)	201 (161, 245)	190 (148, 238.75)	<.001
Triglycerides (mmol/L)	1.20 (0.87, 1.71)	1.21 (0.87, 1.72)	1.17 (0.87, 1.66)	0.007
eGFR (mL/min/1.73m²)	110.90 (92.7, 130.4)	112.3 (94.7, 131.30)	103.40 (78.7, 124.8)	<.001
Surgery-Related Characteristics				
Crystalloid Infusion (ml)	1000 (500, 1100)	1000 (500, 1100)	1000 (500, 1100)	0.696
Colloid Infusion (ml)	500 (500, 1000)	500.00 (500, 1000)	1000 (500, 1000)	<.001
Urine Output (ml)	400 (200, 800)	400 (200, 800)	300.00 (200, 600)	<.001
Intraoperative Blood Loss (ml)	100 (20, 200)	50 (20, 200)	100 (50, 240)	<.001
Total Volume Administered (ml)	1550 (1100, 2100)	1500 (1100, 2100)	1700 (1500, 2400)	<.001
Operative Time (min)	129.60 (72.00, 201.60)	115.2 (72, 187.2)	158.4 (100.8, 230.4)	<.001
Anesthesia Time (min)	144 (100.8, 230.4)	144 (86.4, 216)	187.2 (115.2, 259.2)	<.001
**Intraoperative Medications**				
Dexmedetomidine, n(%)				0.132
No	8219 (35.62%)	6973 (35.82%)	1246 (34.52%)	
Yes	14856 (64.38%)	12492 (64.18%)	2364 (65.48%)	
Norepinephrine, n(%)				<.001
No	15224 (65.98%)	13169 (67.65%)	2055 (56.93%)	

Variables	Total (n = 23075)	Non-AKI group (n = 19465)	AKI group (n = 3610)	*P*
Yes	7851 (34.02%)	6296 (32.35%)	1555 (43.07%)	
Phenylephrine, n(%)				<.001
No	16389 (71.02%)	14114 (72.51%)	2275 (63.02%)	
Yes	6686 (28.98%)	5351 (27.49%)	1335 (36.98%)	
Dopamine, n(%)				0.246
No	16421 (71.16%)	13881 (71.31%)	2540 (70.36%)	
Yes	6654 (28.84%)	5584 (28.69%)	1070 (29.64%)	
Ephedrine, n(%)				0.705
No	13923 (60.34%)	11755 (60.39%)	2168 (60.06%)	
Yes	9152 (39.66%)	7710 (39.61%)	1442 (39.94%)	
Furosemide, n(%)				<.001
No	20334 (88.12%)	17455 (89.67%)	2879 (79.75%)	
Yes	2741 (11.88%)	2010 (10.33%)	731 (20.25%)	
Nicardipine, n(%)				0.043
No	20034 (86.82%)	16862 (86.63%)	3172 (87.87%)	
Yes	3041 (13.18%)	2603 (13.37%)	438 (12.13%)	
Esmolol, n(%)				0.154
No	21557 (93.42%)	18204 (93.52%)	3353 (92.88%)	
Yes	1518 (6.58%)	1261 (6.48%)	257 (7.12%)	
Urapidil, n(%)				0.023
No	21031 (91.14%)	17705 (90.96%)	3326 (92.13%)	
Yes	2044 (8.86%)	1760 (9.04%)	284 (7.87%)	
Atropine, n(%)				0.055
No	15379 (66.65%)	13023 (66.90%)	2356 (65.26%)	
Yes	7696 (33.35%)	6442 (33.10%)	1254 (34.74%)	

Abbreviation: BMI: Body Mass Index, ASA: American Society of Anesthesiologists, ALT: Alanine Aminotransferase, SBP (mmHg): Systolic Blood Pressure, MAP (mmHg): Mean Arterial Pressure.

Table B. Comparison of baseline characteristics between AKD patients and control group.

Variables	Total (n = 7619)	Non-AKD (n = 6480)	AKD (n = 1139)	*P*
**Demographic Characteristic**				
Sex, n(%)				<0.001
Male	4300 (56.44%)	3595 (55.48%)	705 (61.90%)	
Female	3319 (43.56%)	2885 (44.52%)	434 (38.10%)	
Age (Year)	61 (52, 70)	61.00 (52, 69)	63 (54, 71.5)	<0.001
Height (cm)	165 (160, 170)	165 (159, 170)	165 (160, 172)	0.001
Weight (kg)	65 (56, 73)	64.50 (56.0, 72.5)	65.00 (57, 74)	0.006
BMI (kg/m^2)	23.56 (21.23, 26.03)	23.53 (21.22, 25.96)	23.67 (21.43,26.30)	0.177
ASA classification, n (%)				<0.001
I	89 (1.17%)	69 (1.06%)	20 (1.76%)	
II	1135 (14.90%)	996 (15.37%)	139 (12.20%)	
III	4820 (63.26%)	4234 (65.34%)	586 (51.45%)	
IV	1283 (16.84%)	987 (15.23%)	296 (25.99%)	
V	292 (3.83%)	194 (2.99%)	98 (8.60%)	
**Preoperative Comorbidities**				
Hypertension, n(%)				<0.001
No	5121 (67.21%)	4436 (68.46%)	685 (60.14%)	
Yes	2498 (32.79%)	2044 (31.54%)	454 (39.86%)	
Diabetes Mellitus, n(%)				<0.001
No	6679 (87.66%)	5738 (88.55%)	941 (82.62%)	
Yes	940 (12.34%)	742 (11.45%)	198 (17.38%)	
History of Malignancy, n(%)				<0.001
No	4803 (63.04%)	3958 (61.08%)	845 (74.19%)	
Yes	2816 (36.96%)	2522 (38.92%)	294 (25.81%)	
History of Stroke, n(%)				0.002
No	6996 (91.82%)	5977 (92.24%)	1019 (89.46%)	
Yes	623 (8.18%)	503 (7.76%)	120 (10.54%)	
Coronary Artery Disease, n(%)				0.001
No	7186 (94.32%)	6135 (94.68%)	1051 (92.27%)	

Variables	Total<br>(n = 7619)	Non-AKD<br>(n = 6480)	AKD<br>(n = 1139)	*P*
Yes	433 (5.68%)	345 (5.32%)	88 (7.73%)	
Valvular Heart Disease, n(%)				0.498
No	7178 (94.21%)	6100 (94.14%)	1078 (94.64%)	
Yes	441 (5.79%)	380 (5.86%)	61 (5.36%)	
Liver Cirrhosis, n(%)				0.929
No	7503 (98.48%)	6381 (98.47%)	1122 (98.51%)	
Yes	116 (1.52%)	99 (1.53%)	17 (1.49%)	
Anemia, n(%)				0.011
No	7389 (96.98%)	6298 (97.19%)	1091 (95.79%)	
Yes	230 (3.02%)	182 (2.81%)	48 (4.21%)	
**Preoperative Laboratory Tests**				
Albumin (g/L)	39.50 (37.10, 41.50)	39.60 (37.40, 41.70)	38.40 (34.85,40.65)	<0.001
ALT (U/L)	16.9 (11.9, 26.7)	16.7 (11.8, 26.3)	17.5 (12.2, 29.9)	0.018
Creatinine (μmol/L)	62 (51, 75)	61.00 (51, 73.93)	68 (54, 96.50)	<0.001
Direct Bilirubin (μmol/L)	2.20 (1.5, 3.3)	2.10 (1.5, 3.2)	2.40 (1.60, 4)	<0.001
Glucose (mmol/L)	4.85 (4.40, 5.73)	4.81 (4.39, 5.59)	5.16 (4.47, 6.75)	<0.001
Platelet Count (×10^9/L)	199 (156, 247)	200 (158, 247.00)	189 (142, 239)	<0.001
Triglycerides (mmol/L)	1.18 (0.86, 1.66)	1.18 (0.86, 1.66)	1.23 (0.88, 1.69)	0.031
eGFR (mL/min/1.73m^2)	109.90 (90.7, 129.7)	111.30 (93.2, 129.83)	99.5 (66.05, 128.8)	<0.001
**Surgery-Related Characteristics**				
Crystalloid Infusion (ml)	1000 (500, 1100)	1000 (500, 1100)	700.00 (500, 1000)	<0.001
Colloid Infusion (ml)	500 (500, 1000)	550 (500, 1000)	500 (500, 1000)	<0.001
Urine Output (ml)	400 (200, 800)	400 (200, 800)	300 (200, 600)	<0.001
Intraoperative Blood Loss (ml)	100 (20.00, 300.00)	100 (20, 300)	100 (25, 300)	0.009
Total Volume Administered (ml)	1600 (1000, 2500)	1600 (1000, 2500)	1500 (1000, 2450)	0.001
Operative Time (min)	144 (72, 230.4)	158.4 (86.4, 230.4)	129.6 (57.6, 223.2)	<0.001
Anesthesia Time (min)	172.80 (86.4, 259.2)	172.80 (100.8, 259.2)	158.40 (72, 259.2)	<0.001
**Intraoperative Medications**				
Dexmedetomidine, n(%)				0.813
No	2659 (34.90%)	2265 (34.95%)	394 (34.59%)	
Yes	4960 (65.10%)	4215 (65.05%)	745 (65.41%)	

Variables	Total (n = 7619)	Non-AKD (n = 6480)	AKD (n = 1139)	*P*
Norepinephrine, n(%)				<0.001
No	4538 (59.56%)	3954 (61.02%)	584 (51.27%)	
Yes	3081 (40.44%)	2526 (38.98%)	555 (48.73%)	
Phenylephrine, n(%)				<0.001
No	5298 (69.54%)	4571 (70.54%)	727 (63.83%)	
Yes	2321 (30.46%)	1909 (29.46%)	412 (36.17%)	
Dopamine, n(%)				0.010
No	5253 (68.95%)	4505 (69.52%)	748 (65.67%)	
Yes	2366 (31.05%)	1975 (30.48%)	391 (34.33%)	
Ephedrine, n(%)				0.117
No	4588 (60.22%)	3926 (60.59%)	662 (58.12%)	
Yes	3031 (39.78%)	2554 (39.41%)	477 (41.88%)	
Furosemide, n(%)				<0.001
No	6481 (85.06%)	5596 (86.36%)	885 (77.70%)	
Yes	1138 (14.94%)	884 (13.64%)	254 (22.30%)	
Nicardipine, n(%)				0.320
No	6640 (87.15%)	5637 (86.99%)	1003 (88.06%)	
Yes	979 (12.85%)	843 (13.01%)	136 (11.94%)	
Esmolol, n(%)				<0.001
No	6950 (91.22%)	5949 (91.81%)	1001 (87.88%)	
Yes	669 (8.78%)	531 (8.19%)	138 (12.12%)	
Urapidil, n(%)				0.402
No	6830 (89.64%)	5801 (89.52%)	1029 (90.34%)	
Yes	789 (10.36%)	679 (10.48%)	110 (9.66%)	
Atropine, n(%)				0.260
No	5041 (66.16%)	4304 (66.42%)	737 (64.71%)	
Yes	2578 (33.84%)	2176 (33.58%)	402 (35.29%)	

Abbreviation: BMI: Body Mass Index, ASA: American Society of Anesthesiologists, ALT: Alanine Aminotransferase, SBP (mmHg): Systolic Blood Pressure, MAP (mmHg): Mean Arterial Pressure.

Table C. Summary of Data Completeness and Outlier Detection for the Internal Dataset (Nanjing Drum Tower Hospital, 2013–2023)

Feature	Total samples	Missing values (n, %)	Abnormal values (n, %)	Valid values (%)
T	236150299	85088128 (36.03%)	127525 (0.05%)	63.91%
HEART_RATE	236150299	23256043 (9.85%)	3105 (0)	90.15%
BREATH	236150299	28367373 (12.01%)	274885 (0.12%)	87.87%
PULSE	236150299	21534983 (9.12%)	39953 (0.02%)	90.86%
SPO_2	236150299	21715685 (9.2%)	15349 (0.01%)	90.80%
NON_INVASIVE_SBP	236150299	61452192 (26.02%)	20094 (0.01%)	73.97%
NON_INVASIVE_DBP	236150299	61540304 (26.06%)	25166 (0.01%)	73.93%
PSBP	236150299	102649065 (43.47%)	302713 (0.13%)	56.40%
PDBP	236150299	102686069 (43.48%)	369654 (0.16%)	56.36%
MAP	236150299	102207318 (43.28%)	784477 (0.33%)	56.39%
CVP	236150299	182815102 (77.41%)	2390435 (1.01%)	21.57%
$ETCO_2$	236150299	58730783 (24.87%)	1028258 (0.44%)	74.69%

Abbreviations:

- T: Body temperature during operation
- HEART_RATE: Heart rate (beats per minute, bpm)
- BREATH: Respiratory rate (breaths per minute)
- PULSE: Pulse rate (beats per minute)
- SPO_2: Peripheral oxygen saturation (%)
- NON_INVASIVE_SBP: Non-invasive systolic blood pressure (mmHg)
- NON_INVASIVE_DBP: Non-invasive diastolic blood pressure (mmHg)

- PSBP: Pulse-derived systolic blood pressure (mmHg)
- PDBP: Pulse-derived diastolic blood pressure (mmHg)
- MAP: Mean arterial pressure (mmHg)
- CVP: Central venous pressure (mmHg)
- $ETCO_2$: End-tidal carbon dioxide (mmHg)

Table D. Summary of Data Completeness and Outlier Detection for the External Validation Dataset (VitalDB, South Korea)

Feature	Total samples	Missing values (n, %)	Abnormal values (n, %)	Valid values (%)
T	2,015,352	392,719 (19.5%)	624 (0.03%)	80.5%
HEART_RATE	2,015,352	113,343 (5.6%)	3,328 (0.17%)	94.2%
BREATH	2,015,352	202,367 (10.0%)	24,848 (1.2%)	88.7%
PULSE	2,015,352	80,996 (4.0%)	1,005 (0.05%)	95.9%
SPO_2	2,015,352	80,807 (4.0%)	344 (0.02%)	96.0%
NON_INVASIVE_SBP	2,015,352	1,077,907 (53.5%)	63 (0.00%)	46.5%
NON_INVASIVE_DBP	2,015,352	1,077,908 (53.5%)	57 (0.00%)	46.5%
PSBP	2,015,352	753,140 (37.4%)	66,422 (3.3%)	59.3%
PDBP	2,015,352	752,956 (37.4%)	71,895 (3.6%)	59.1%
MAP	2,015,352	744,987 (37.0%)	78,780 (3.9%)	59.1%
CVP	2,015,352	1,500,292 (74.4%)	106,159 (5.3%)	20.3%
$ETCO_2$	2,015,352	167,925 (8.3%)	53,976 (2.7%)	89.0%

Abbreviations: Same as in Table S3.

Table E. Transformer Model Architecture, Hyperparameters, and Training Configuration

Category	Parameter	Setting / Description	Rationale / Notes
Model Overview	Model type	Transformer-based deep learning classifier	Used for sequence modeling of time-series vital signs
	Prediction horizons	5, 10, and 15 minutes	Represent actionable clinical windows for IOH prevention
	Input sequence length	15 timepoints (1-min intervals)	Captures recent physiological trends
	Input features	17 dynamic + 2 static features	Routine intraoperative vital signs
Architecture Parameters	Encoder layers	8	Balances model capacity and computational cost
	Attention heads	8	Captures multiple temporal dependency patterns
	Model dimension (*d*model)	64	Sufficient for capturing complex signals without overfitting
	Feed-forward dimension	256	Standard ratio (≈4× *d*model)
	Dropout rate	0.1	Prevents overfitting
	Positional encoding	Sinusoidal	Enables temporal order awareness

Category	Parameter	Setting / Description	Rationale / Notes
Training Parameters	Optimizer	AdamW	Effective for large-scale transformer training
	Learning rate	2×10^{-4}	Tuned via grid search
	Batch size	400–1000	Adjusted based on prediction horizon
	Epochs	200	Ensures convergence
	Loss function	Combined Focal Loss + AUC-ranking loss	Enhances recall and discriminative performance
	Focal Loss parameters	$\alpha = 0.6$, $\gamma = 2$	Addresses class imbalance and hard samples
	AUC loss weight	0.2	Optimizes ranking quality
	Positive class weight	2.0	Compensates for label imbalance
	Early stopping	Patience = 20 epochs	Prevents overtraining
Data Configuration	Sampling interval	1 minute	Uniform across all variables
	Train/validation split	80% / 20% by patient ID	Prevents patient-level leakage
	Label definition	MAP < 65 mmHg for ≥1 minute	Clinically validated IOH threshold
Calibration	Calibration methods	Isotonic regression and Platt scaling	Improves probability calibration (ECE)

Category	Parameter	Setting / Description	Rationale / Notes
Hardware / Runtime	Hardware	NVIDIA GeForce RTX 3080 GPU	10 GB VRAM, CUDA acceleration
	Training time	~18 hours per model	Average wall time per horizon
	Memory usage	8–12 GB	During data preprocessing and training
Evaluation Metrics	Primary metric	AUROC	Measures discriminative performance
	Secondary metrics	Accuracy, F1, Recall, Specificity, ECE	Evaluates calibration and clinical utility

Detailed configuration of the Transformer-based models used to predict intraoperative hypotension (IOH) at 5-, 10-, and 15-minute horizons. Each model shared the same architecture and hyperparameters except for minor differences in batch size. The loss function combined Focal Loss (to address class imbalance) and an AUC-ranking term (to enhance discrimination). Model calibration was performed using isotonic regression and Platt scaling.

Abbreviations:

MAP: Mean arterial pressure

IOH: Intraoperative hypotension

ECE: Expected calibration error

AUROC: Area under the receiver operating characteristic curve

GPU: Graphics processing unit

Table F. Computational Requirements for Transformer and XGBoost Models

Category	Transformer Model	XGBoost Model
Hardware for training	NVIDIA GeForce RTX 3080 (10 GB VRAM), Intel i9 CPU, 64 GB RAM	Apple M2 Ultra CPU, 196 GB RAM
Software environment	Python 3.12, PyTorch 2.2, scikit-learn, pandas, numpy	Python 3.12, XGBoost 2.1.0, scikit-learn, pandas, numpy
Training duration (per model)	~18 hours (GPU accelerated)	~10–30 minutes (CPU)
Memory usage during training	8–12 GB	~8 GB
Inference time (per case)	<100 ms	<10 ms
Model size	~200 MB	~500 MB
Hardware requirement for inference	GPU optional; real-time compatible on standard CPU	CPU only; real-time compatible
Batch prediction capability	Supported (parallelized inference)	Supported (CPU multithreading)

Both models were optimized for real-time inference during surgery. The Transformer model benefits from GPU acceleration during training but can be deployed efficiently on CPUs for live intraoperative use. The XGBoost model requires no specialized hardware and achieves extremely fast inference (<10 ms), making it suitable for integration into bedside decision-support systems.

Table G. Performance of the XGBoost and Transformer Model in High-Risk Surgical Subgroups (Cardiac, Thoracic, and Vascular Surgeries)

Model	Accuracy	F1 Score	Precision	Recall	Specificity	Sensitivity
**XGBoost 5min**	0.9081	0.7312	0.7137	0.7495	0.9399	0.7495
**XGBoost 10min**	0.8864	0.6567	0.6615	0.6521	0.9333	0.6521
**XGBoost 15min**	0.8779	0.6223	0.642	0.6038	0.9327	0.6038

	AUC (95% CI)	ECE (95% CI)	Support
**XGBoost 5min**	0.9354 (0.9348, 0.9356)	0.1242 (0.1238, 0.1244)	1,104,780 (1=184,130.0, 0=920,650.0)
**XGBoost 10min**	0.9023 (0.9017, 0.9025)	0.1189 (0.1188, 0.1191)	1,080,666 (1=180,111.0, 0=900,555.0)
**XGBoost 15min**	0.8865 (0.8861, 0.8869)	0.1135 (0.1134, 0.1138)	1,047,834 (1=174,639.0, 0=873,195.0)

Model	Accuracy	F1 Score	Precision	Recall	Specificity	Sensitivity
**Transformer 5min**	0.7824	0.6481	0.4869	0.9042	0.7215	0.9042
**Transformer 10min**	0.7613	0.6235	0.4627	0.8921	0.7048	0.8921
**Transformer 15min**	0.7329	0.5968	0.4398	0.8834	0.6812	0.8834

	AUC (95% CI)	ECE (95% CI)	Support
**Transformer 5min**	0.9216 (0.9209, 0.9221)	0.0418 (0.0412, 0.0423)	1,104,780 (1=184,130; 0=920,650)
**Transformer 10min**	0.8958 (0.8951, 0.8964)	0.0386 (0.0381, 0.0392)	1,080,666 (1=180,111; 0=900,555)
**Transformer 15min**	0.8739 (0.8732, 0.8746)	0.0352 (0.0347, 0.0358)	1,047,834 (1=174,639; 0=873,195)

Table H. Sensitivity Analysis of XGBoost and Transformer Models Under an Alternative IOH Definition (MAP < 55 mmHg)

Model	Accuracy	F1 Score	Precision	Recall	Specificity	Sensitivity
**XGBoost 5min**	0.3843	0.3491	0.2119	0.9906	0.2631	0.9906
**XGBoost 10min**	0.3405	0.3338	0.2007	0.9915	0.2103	0.9915
**XGBoost 15min**	0.3479	0.3354	0.202	0.9875	0.22	0.9875

	AUC (95% CI)	ECE (95% CI)	Support
**XGBoost 5min**	0.9262 (0.9242, 0.9279)	0.1319 (0.1315, 0.1332)	216,366 (1=36,061.0, 0=180,305.0)
**XGBoost 10min**	0.9055 (0.905, 0.9069)	0.1284 (0.1281, 0.1291)	208,716 (1=34,786.0, 0=173,930.0)
**XGBoost 15min**	0.8930 (0.8921, 0.8935)	0.1253 (0.1247, 0.1255)	199,944 (1=33,324.0, 0=166,620.0)

Model	Accuracy	F1 Score	Precision	Recall	Specificity	Sensitivity
**Transformer 5min**	0.8958	0.7061	0.81	0.6258	0.9633	0.6258
**Transformer 10min**	0.8686	0.6787	0.6641	0.694	0.9122	0.694
**Transformer 15min**	0.8413	0.4695	0.7082	0.3511	0.9638	0.3511

	AUC (95% CI)	ECE (95% CI)	Support
**Transformer 5min**	0.8977 (0.8955, 0.8998)	0.0028 (0.0020, 0.0044)	155,099 (1=31,020, 0=124,079)
**Transformer 10min**	0.8632 (0.8576, 0.8686)	0.0516 (0.0489, 0.0548)	145,330 (1=29,066, 0=116,264)
**Transformer 15min**	0.7318 (0.7283, 0.7357)	0.0244 (0.0226, 0.0263)	137,975 (1=27,595, 0=110,380)

Table I. Sensitivity Analysis of XGBoost and Transformer Models Under an Alternative IOH Definition (MAP < 60 mmHg)

Model	Accuracy	F1 Score	Precision	Recall	Specificity	Sensitivity
**XGBoost 5min**	0.4211	0.363	0.2222	0.9895	0.3074	0.9895
**XGBoost 10min**	0.3806	0.3473	0.2106	0.9886	0.259	0.9886
**XGBoost 15min**	0.3742	0.3447	0.2088	0.9877	0.2515	0.9877
**Transformer 5min**	0.8967	0.7186	0.7892	0.6597	0.956	0.6597
**Transformer 10min**	0.8023	0.4988	0.5059	0.4919	0.8799	0.4919
**Transformer 15min**	0.7556	0.4637	0.4132	0.5282	0.8125	0.5282

Model	AUC (95% CI)	ECE (95% CI)	Support
**XGBoost 5min**	0.9284 (0.9279, 0.9293)	0.1308 (0.1305, 0.1313)	429,078 (1=71,513.0, 0=357,565.0)
**XGBoost 10min**	0.9006 (0.9002, 0.9009)	0.1260 (0.1254, 0.1263)	417,114 (1=69,519.0, 0=347,595.0)
**XGBoost 15min**	0.8869 (0.8863, 0.8875)	0.1230 (0.1221, 0.1232)	401,784 (1=66,964.0, 0=334,820.0)
**Transformer 5min**	0.9123 (0.9110, 0.9137)	0.0014 (0.0013, 0.0027)	327,794 (1=65,559, 0=262,235)
**Transformer 10min**	0.7784 (0.7746, 0.7819)	0.0553 (0.0528, 0.0574)	303,535 (1=60,707, 0=242,828)
**Transformer 15min**	0.7474 (0.7437, 0.7511)	0.0714 (0.0689, 0.0740)	285,060 (1=57,012, 0=228,048)

Table J. Performance of XGBoost and Transformer models after interpolation-based preprocessing of non-invasive blood pressure measurements for 5-minute IOH prediction in internal and external validation datasets

Model	Accuracy	F1 Score	Precision	Recall	Specificity	Sensitivity
**XGBoost (Internal)**	0.8903	0.7387	0.7057	0.7749	0.9192	0.7749
**XGBoost (Vitaldb)**	0.7874	0.6095	0.4816	0.8297	0.7768	0.8297
**Transformer (Internal)**	0.8935	0.728	0.744	0.7126	0.9387	0.7126
**Transformer (Vitaldb)**	0.7185	0.2329	0.256	0.2136	0.8448	0.2136

Model	AUC (95% CI)	ECE (95% CI)	Support
**XGBoost (Internal)**	0.9312 (0.9310, 0.9316)	0.1170 (0.1168, 0.1175)	927,111 (1=185,422-0=741,689)
**XGBoost (Vitaldb)**	0.8777 (0.8764, 0.8792)	0.1542 (0.1527, 0.1553)	262,545 (1=52,509-0=210,036)
**Transformer (Internal)**	0.9224 (0.9217, 0.9230)	0.0009 (0.0007, 0.0016)	927,111 (1=185,422-0=741,689)
**Transformer (Vitaldb)**	0.6352 (0.6327, 0.6374)	0.1364 (0.1350, 0.1381)	262,545 (1=52,509-0=210,036)

Table K. Subgroup Performance of the XGBoost Model for 5-Minute Intraoperative Hypotension Prediction Across Age and ASA Risk Categories in Internal and External Validation Cohorts.

Dataset	Accuracy	F1 Score	Precision	Recall	Specificity	Sensitivity
Internal(Baseline)	0.8896	0.7376	0.7031	0.7758	0.9181	0.7758
Internal(Age<65)	0.8899	0.7384	0.7043	0.7759	0.9184	0.7759
Internal(Age>=65)	0.8897	0.7385	0.7044	0.776	0.9182	0.776
Internal(ASA<=II)	0.8903	0.7387	0.7034	0.7778	0.9183	0.7778
Internal(ASA >=III)	0.8897	0.7379	0.7021	0.7775	0.9177	0.7775
Vitaldb(Baseline)	0.7861	0.6085	0.4799	0.8312	0.7748	0.8312
Vitaldb(Age<65)	0.7845	0.6069	0.4785	0.8295	0.7732	0.8295
Vitaldb(Age>=65)	0.7852	0.608	0.4791	0.8319	0.7735	0.8319
Vitaldb(ASA<=II)	0.7856	0.6084	0.4795	0.8323	0.7739	0.8323
Vitaldb(ASA >=III)	0.786	0.6079	0.4787	0.8325	0.7744	0.8325

	AUC (95% CI)	ECE (95% CI)
Internal(Baseline)	0.9305 (0.9301, 0.9309)	0.1201 (0.1198, 0.1204)
Internal(Age<65)	0.9303 (0.9299, 0.9304)	0.1204 (0.1201, 0.1206)
Internal(Age>=65)	0.9307 (0.9302, 0.9314)	0.1202 (0.1198, 0.1204)
Internal(ASA<=II)	0.9311 (0.9305, 0.9318)	0.1207 (0.1201, 0.1217)
Internal(ASA >=III)	0.9307 (0.9301, 0.9314)	0.1202 (0.1199, 0.1205)
Vitaldb(Baseline)	0.8773 (0.8753, 0.8784)	0.1548 (0.1537, 0.1555)
Vitaldb(Age<65)	0.8754 (0.8734, 0.8774)	0.1547 (0.1542, 0.1559)
Vitaldb(Age>=65)	0.8777 (0.8757, 0.8786)	0.1551 (0.1551, 0.1566)
Vitaldb(ASA<=II)	0.8769 (0.8758, 0.8798)	0.1550 (0.1540, 0.1552)
Vitaldb(ASA >=III)	0.8768 (0.8750, 0.8774)	0.1555 (0.1550, 0.1567)

Table L. Subgroup Performance of the Transformer Model for 5-Minute Intraoperative Hypotension Prediction Across Age and ASA Risk Categories in Internal and External Validation Cohorts.

Dataset	Accuracy	F1 Score	Precision	Recall	Specificity	Sensitivity
Internal(Baseline)	0.8934	0.7262	0.748	0.7057	0.9404	0.7057
Internal(ASA<=II)	0.8935	0.7256	0.7482	0.7044	0.9408	0.7044
Internal(Age>=65)	0.8934	0.7274	0.7429	0.7125	0.9385	0.7125
Internal(ASA >=III)	0.8936	0.726	0.7469	0.7063	0.9403	0.7063
Internal(Age<65)	0.8931	0.7265	0.7453	0.7087	0.9393	0.7087
Vitaldb(Baseline)	0.8333	0.6343	0.5633	0.7257	0.8601	0.7257
Vitaldb(ASA<=II)	0.8325	0.632	0.5656	0.716	0.8618	0.716
Vitaldb(Age>=65)	0.8325	0.6327	0.5637	0.7208	0.8605	0.7208
Vitaldb(ASA >=III)	0.8322	0.6332	0.5644	0.7209	0.8601	0.7209
Vitaldb(Age<65)	0.8322	0.6321	0.5636	0.7196	0.8604	0.7196

	AUC (95% CI)	ECE (95% CI)
Internal(Baseline)	0.9219 (0.9209, 0.9229)	0.0007 (0.0007, 0.0018)
Internal(ASA<=II)	0.9220 (0.9211, 0.9230)	0.0010 (0.0009, 0.0021)
Internal(Age>=65)	0.9224 (0.9215, 0.9234)	0.0012 (0.0009, 0.0022)
Internal(ASA >=III)	0.9221 (0.9212, 0.9231)	0.0008 (0.0008, 0.0019)
Internal(Age<65)	0.9221 (0.9212, 0.9231)	0.0008 (0.0008, 0.0020)
Vitaldb(Baseline)	0.8809 (0.8788, 0.8833)	0.0573 (0.0554, 0.0590)
Vitaldb(ASA<=II)	0.8784 (0.8759, 0.8804)	0.0563 (0.0546, 0.0581)
Vitaldb(Age>=65)	0.8792 (0.8771, 0.8814)	0.0563 (0.0545, 0.0581)
Vitaldb(ASA >=III)	0.8793 (0.8770, 0.8816)	0.0559 (0.0540, 0.0577)
Vitaldb(Age<65)	0.8794 (0.8771, 0.8817)	0.0560 (0.0543, 0.0577)

Supplemental Figures

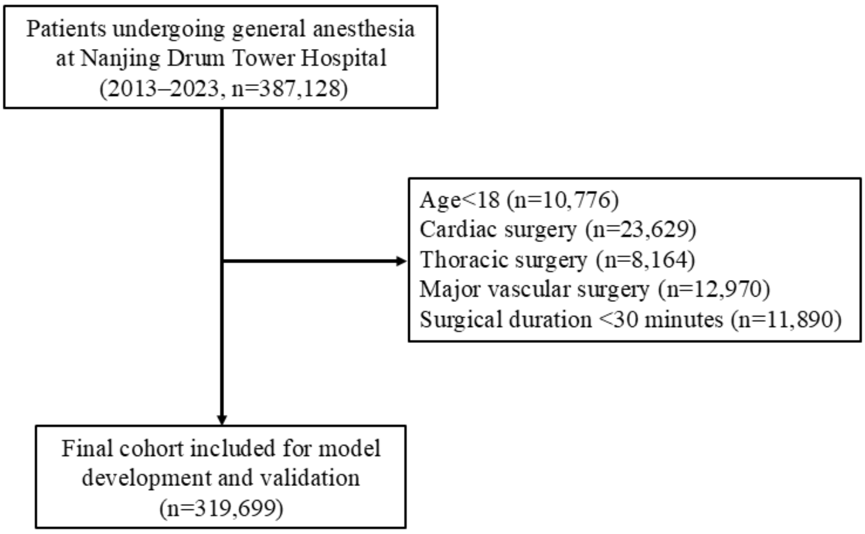

Figure A. Flowchart showing patient selection for the model development cohort at Nanjing Drum Tower Hospital.

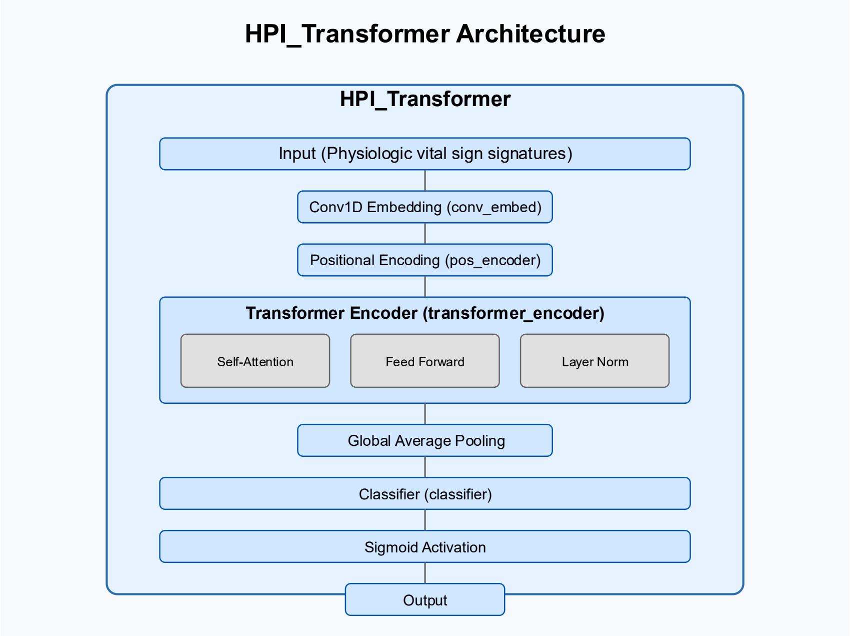

Figure B. HPI transformer architecture.

Each of the three Transformer classifiers (5--10--and 15-minute prediction horizons) employed an 8-layer encoder with 8 attention heads-a model dimension (d_{model}) of 64-feed-forward dimension 256-and dropout 0.1. Models were trained for 200 epochs using the AdamW optimizer (learning rate = 2×10^{-4}-batch size = 400–1000) with a combined Focal Loss (α = 0.6-γ = 2) and AUC-ranking loss. Full details-including feature definitions-tuning parameters-and hardware configuration-are provided in Supplementary Table S5.

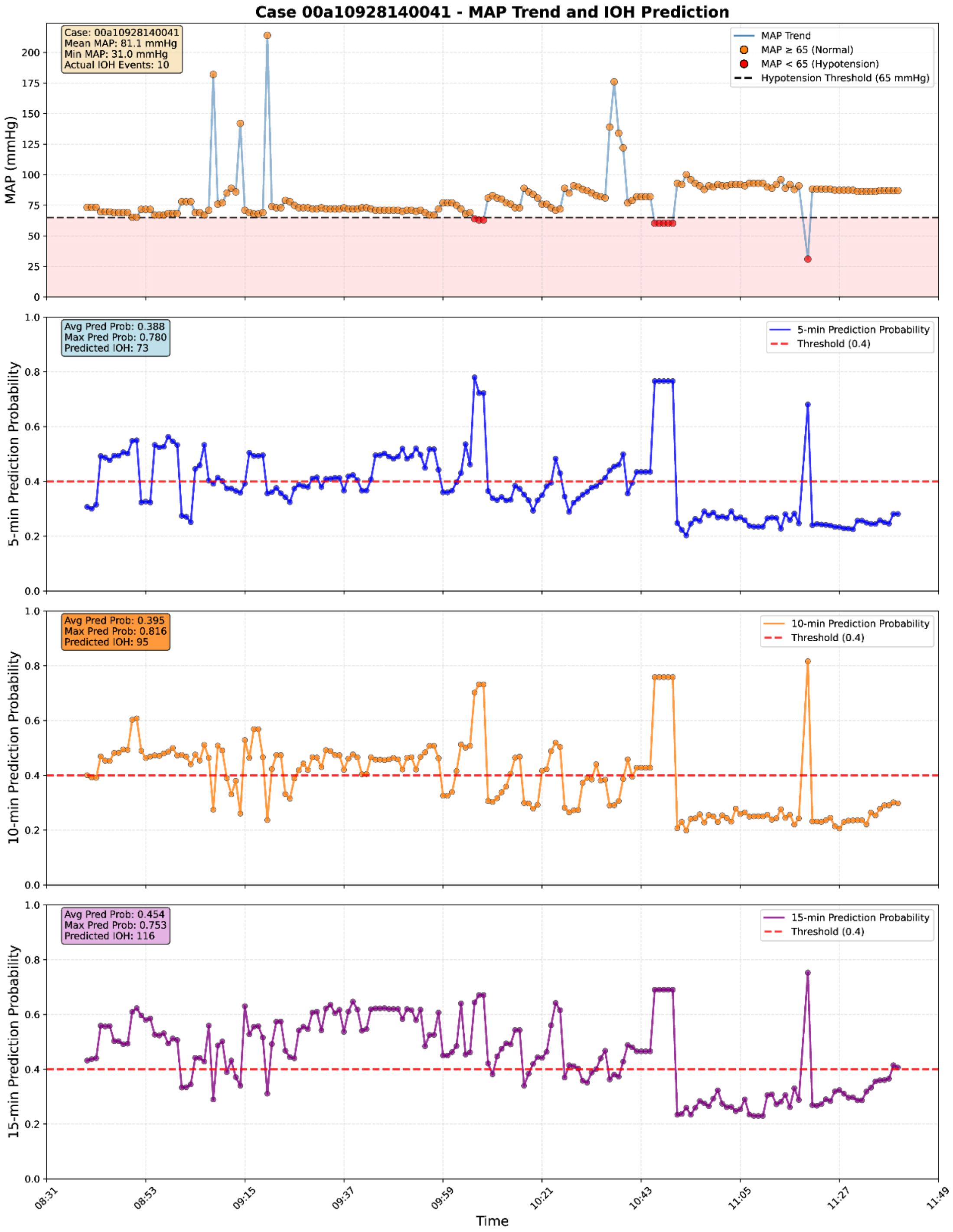

Figure C. Intraoperative mean arterial pressure (MAP) trajectories and Transformer-based predictions of intraoperative hypotension (IOH) risk in a representative surgical case

The top panel displays the intraoperative mean arterial pressure (MAP) trajectory-where the solid blue line represents continuous MAP measurements. Orange circles indicate normotensive states (MAP ≥ 65 mmHg)-while red circles denote hypotensive episodes (MAP < 65 mmHg). The black dashed horizontal line marks the clinical

hypotension threshold at 65 mmHg-with shaded backgrounds highlighting normotensive (light orange) and hypotensive (light red) ranges. The lower panels show Transformer-based predicted intraoperative hypotension (IOH) risk probabilities at 5--10--and 15-minute prediction horizons-represented by blue-orange-and purple lines-respectively. Red dashed lines in each prediction panel indicate the predefined risk threshold. Insets summarize the mean and maximum predicted probabilities-as well as the total number of predicted IOH events exceeding the threshold.

Supplemental Method

Method A. Preprocessing and Standardization of the VitalDB Dataset

To ensure consistency between the external VitalDB dataset and the internal cohort from Nanjing Drum Tower Hospital-we applied a harmonized preprocessing and standardization pipeline. The VitalDB dataset (Lee et al.-Scientific Data-2022) includes high-resolution intraoperative physiological data from a tertiary hospital in South Korea. For the present study-we selected adult patients (⩾18 years) who underwent non-cardiac surgery under general anesthesia. Cardiac-thoracic-and major vascular procedures were excluded because of their distinct hemodynamic characteristics and frequent use of invasive monitoring. Cases with insufficient surgical duration (<30 minutes) or with more than 10% missing vital sign values were also excluded to ensure data completeness and temporal continuity.

The following physiological variables were extracted from VitalDB: mean arterial pressure (MAP)-systolic and diastolic blood pressure (SBP and DBP)-heart rate (HR)-peripheral oxygen saturation (SpO_2)-respiratory rate (RR)-and end-tidal carbon dioxide ($ETCO_2$). Variable names and units were standardized to match the internal dataset schema. All variables were resampled to a one-minute interval to ensure temporal alignment across parameters. For non-invasive blood pressure-which was typically recorded at three- to five-minute intervals-values were forward-filled until the next available measurement-consistent with standard clinical charting practices. Implausible outliers (e.g.-MAP <30 mmHg or >200 mmHg-HR <25 bpm or >220 bpm) were removed-and remaining extreme values were truncated at the 0.1st and 99.9th percentiles to mitigate the influence of artifacts.

Each physiological variable was then standardized using z-score normalization-with the mean and standard deviation calculated from the VitalDB population. This normalization ensured that variable scales were comparable between datasets and prevented bias during external validation. Finally-all time-series were synchronized by timestamp-producing a uniform multivariate matrix structure identical to the internal

dataset format. The resulting dataset was stored in comma-separated value (CSV) files for analysis.

This harmonized preprocessing pipeline allowed the external VitalDB data to be directly compatible with the model trained on the internal dataset-minimizing distributional drift and ensuring a valid assessment of model generalizability.

Methods B. XGBoost Model Configuration and Hyperparameter Tuning

The XGBoost classifier was developed as a benchmark model for comparison with the Transformer-based architecture. It was trained to predict intraoperative hypotension (IOH-defined as mean arterial pressure [MAP] < 65 mmHg for ≥ 1 minute) at 5--10--and 15-minute horizons using the same cleaned and standardized intraoperative vital sign dataset as the Transformer models.

Input Features

The XGBoost model used a total of **32 numerical input features**-consisting of 12 basic physiological variables and 20 derived hemodynamic indices.

(A) Basic physiological features (n = 12)

HEART_RATE (beats per minute)-MAP (mmHg)-CVP (central venous pressure-mmHg)-$ETCO_2$ (end-tidal carbon dioxide-mmHg)-PULSE-BREATH (respiratory rate-per minute)-SPO_2 (oxygen saturation-%)-PDBP (diastolic blood pressure-mmHg)-PSBP (systolic blood pressure-mmHg)-NON_INVASIVE_SBP-NON_INVASIVE_DBP-and T (temperature-°C).

(B) Derived dynamic and physiologic indices (n = 20)

To capture short-term variability and hemodynamic stability-20 derived features were computed-including:

- **MSI (Myocardial Stress Index)** = HEART_RATE / MAP
- **PPG (Perfusion Pressure Gradient)** = MAP − CVP
- **PTT (Pulse Interval–Time Index)** = (1 / PULSE) × (PDBP / PSBP)
- **HRI (Cardio-Respiratory Integrated Index)** = (PSBP − PDBP) / HEART_RATE × BREATH
- **SHOCK (Modified Shock Index)** = MAP / PSBP

The derived indices used in the XGBoost model were constructed based on established hemodynamic and cardiorespiratory concepts reported in prior literature-and were adapted to the context of minute-level intraoperative monitoring. These indices are not intended as novel physiological markers-but rather as pragmatic summary features reflecting perfusion status-vascular tone-and cardiorespiratory interaction. For example-MSI (heart rate / MAP) has been used as a bedside prognostic tool correlated with clinical severity and outcomes in emergency and critical care settings[1]. Perfusion pressure gradients (PPG)-calculated as the difference between mean arterial pressure and central venous pressure-represent the driving force for organ blood flow and have been described widely in hemodynamic physiology literature[2]. Pulse transit time (PTT) has also been investigated in prior work as a surrogate measure related to blood pressure dynamics[3]. Cardiorespiratory integrated features (e.g.-HRI) were inspired by existing work on the interaction between heart rate and respiratory signals[4]. Shock-related indices (e.g.-Modified Shock Index variants) have been evaluated in prior clinical studies of hemodynamic instability and outcome prediction[5]

- Short-term dynamics of MAP-HR-and BP-including:
    - 5-minute change rates (e.g.-MAP_CR5M-HR_RATE_CR5M)
    - 5-minute mean/negative slopes (MAP_MNS5M)
    - Trend direction over 3 minutes (MAP_TD3M)
    - 5-minute moving standard deviation (MAP_ST5M)
    - Ratios and rate-of-change indices (MAP2HR_CR5M-MAP2HR_EWM-PP_MAP_RC5M).

These features were chosen to reflect perfusion stability-vascular tone-and cardiorespiratory interaction over short temporal windows.

Training Parameters

Three independent models were trained corresponding to the 5--10--and 15-minute prediction horizons.

- Objective function: binary:logistic with Focal Loss
- Evaluation metric: AUC
- Learning rate: 0.02

- Gamma: 0.1
- Positive-to-negative sample ratio: 1:4
- Maximum depth: 30 (5 min)-45 (10 min)-90 (15 min)
- Number of boosting rounds: 300 (5 min)-400 (10 min)-500 (15 min)
- Early stopping: 100 rounds
- Cross-validation: 5-fold grid search
- Scale_pos_weight adjusted to correct class imbalance

Training and Evaluation

- **Hardware**: Apple M2 Ultra-196 GB RAM-Python 3.12-XGBoost v2.1.0
- **Training set**: ~3.7 million records
- **Validation set**: ~0.9 million records
- **Training time**: 10–30 minutes per model
- **Performance metrics:**
    - ROC-AUC = 0.905 (5 min)-0.892 (10 min)-0.882 (15 min) [internal dataset]
    - ECE (range): 0.12 – 0.21
    - External validation (VitalDB): AUC = 0.901 (5 min).

Hyperparameter Tuning

Hyperparameter tuning was performed using a systematic grid search combined with 5-fold cross-validation on the training dataset to optimize the area under the ROC curve (AUC) and expected calibration error (ECE).

The following parameters were included in the search space:

- **max_depth: 10–100 (step = 10)**
- **n_estimators: 200–600 (step = 50)**
- **learning_rate: 0.01–0.05 (step = 0.01)**
- **gamma: 0.05–0.3 (step = 0.05)**
- **subsample: 0.6–1.0 (step = 0.1)**
- **scale_pos_weight: 1–8 (integer step = 1)**
- **colsample_bytree: 0.6–1.0 (step = 0.1)**

The optimal hyperparameter set for each model (5--10--and 15-minute prediction horizons) was determined by maximizing validation AUC while maintaining calibration stability across folds. Early stopping (100 rounds) was applied to prevent overfitting.

Reference:

[1] Hamade B-Bayram J D-Hsieh Y H-et al. Modified Shock Index as a Predictor of Admission and In-hospital Mortality in Emergency Departments; an Analysis of a US National Database [J]. Arch Acad Emerg Med-2023-11(1): e34.

[2] Caplan M-Chew M S-Hamzaoui O. Central venous pressure: current uses and prospects for an old parameter [J]. Intensive Care Medicine-2025-51(7): 1363-6.

[3] Feng J-Huang Z-Zhou C-et al. Study of continuous blood pressure estimation based on pulse transit time-heart rate and photoplethysmography-derived hemodynamic covariates [J]. Australas Phys Eng Sci Med-2018-41(2): 403-13.

[4] Scholkmann F-Wolf U. The Pulse-Respiration Quotient: A Powerful but Untapped Parameter for Modern Studies About Human Physiology and Pathophysiology [J]. Front Physiol-2019-10: 371.

[5] Liu Y C-Liu J H-Fang Z A-et al. Modified shock index and mortality rate of emergency patients [J]. World J Emerg Med-2012-3(2): 114-7.
