# Supplementary material for: Transformer-based deep learning model for real-time prediction of intraoperative hypotension using dynamic time-series vital signs: A retrospective study
Source: PLoS Med. 2026 Mar 25;23(3):e1005024. doi: 10.1371/journal.pmed.1005024 (PMC13046278; doi:10.1371/journal.pmed.1005024)
Supplement: S1 Study Protocol — (DOC) [file pmed.1005024.s002.doc]

**Supplementary File S3. Study Protocol**

**Title:** *Development and External Validation of a Transformer-Based Model for Real-Time Prediction of Intraoperative Hypotension Using Dynamic Time-Series Vital Signs*

**Principal Investigator:**
Dr. Xiaoping Gu
Department of Anesthesiology
Nanjing Drum Tower Hospital, Affiliated Hospital of Medical School, Nanjing University

**Protocol Version:** 1.0
**Approval Number:** 2025-0157-01
**Approval Date:** March 6, 2025

**1. Background and Rationale**

Intraoperative hypotension (IOH) is a common and clinically significant phenomenon during general anesthesia, associated with increased risks of acute kidney injury (AKI), myocardial injury, and prolonged hospital stays. Current prediction tools are often limited by the need for invasive waveform data. This study aims to develop a Transformer-based deep learning model using routinely collected intraoperative vital signs for real-time prediction of IOH, thereby enabling more accessible and generalizable clinical decision support.

**2. Objectives**

- **Primary Objective:**
  To develop and validate a deep learning model (HPI_Transformer) capable of predicting intraoperative hypotension (MAP <65 mmHg sustained for ≥1 minute) using only continuous time-series vital signs.
- **Secondary Objectives:**
  - To assess model performance at 5-, 10-, and 15-minute prediction horizons.
  - To conduct external validation using an independent dataset (VitalDB).
  - To simulate operational deployment and evaluate clinical alerting performance.
  - To assess the association between IOH burden and postoperative AKI/AKD.

**3. Study Design**

This is a retrospective observational study utilizing a large cohort of surgical patients (n=319,699) who received general anesthesia between January 1, 2013, and December 30, 2023, at Nanjing Drum Tower Hospital. A nested cohort analysis will evaluate the clinical impact of IOH burden on postoperative outcomes.

**4. Ethical Considerations**

This study protocol was reviewed and approved by the Ethics Committee of Nanjing Drum Tower Hospital, affiliated with Nanjing University Medical School (Approval No. 2025-0157-01). Due to the retrospective nature of the study and the use of de-identified data, the requirement for informed consent was waived. No deviations from the approved protocol will be permitted without prior approval.

**5. Study Population**

- **Inclusion Criteria:**
  - Adult patients (≥18 years)
  - Underwent general anesthesia for elective or emergency surgical procedures
- **Exclusion Criteria:**
  - Cardiac, thoracic, or major vascular surgeries
  - Incomplete intraoperative monitoring data
  - Surgical duration insufficient for model input and prediction windows

**6. Data Sources and Variables**

- **Vital Signs Collected:** Heart rate, pulse rate, systolic and diastolic blood pressure, MAP, respiratory rate, SpO₂, ETCO₂
- **Data Frequency:** Aligned to 1-minute intervals; non-invasive BP readings forward-filled
- **Outcome Definition:** IOH defined as MAP <65 mmHg lasting ≥1 minute
- **AKI/AKD Defined According to:** KDIGO and ADQI criteria

**7. Model Development**

A Transformer-based neural network will be constructed using the PyTorch framework. Input includes 10-minute sequences of vital signs to predict hypotension 5, 10, or 15 minutes in advance. Conv1D layers, positional encoding, multi-head attention, and a feedforward decoder constitute the core architecture. Performance will be compared to an XGBoost baseline.

**8. Model Evaluation**

Metrics include AUC-ROC, AUC-PR, precision, recall, F1 score, specificity, and expected calibration error (ECE). Internal validation (20% holdout) and external validation using VitalDB (n=6,388) will be conducted.

**9. Operational Deployment Simulation**

An alert system using a 5-minute lockout and second-stage filtering will be tested for practical utility. Alert sensitivity, frequency, and PPV will be assessed.

**10. Statistical Analysis**

Multivariable logistic regression will evaluate the association between IOH burden and postoperative AKI/AKD, adjusting for patient demographics, comorbidities, and intraoperative variables. Analyses will be performed using R and Python. A formal sample size calculation was not required due to the large dataset.

**11. Data Availability and Security**

All data are securely stored within the hospital’s protected research environment. External datasets (VitalDB) are open-access. Patient confidentiality is strictly maintained; no personally identifiable information is included in the model.
